# A systematic review on integrated care for traumatic brain injury, mental health, and substance use

**DOI:** 10.1371/journal.pone.0264116

**Published:** 2022-03-03

**Authors:** Vincy Chan, Danielle Toccalino, Samira Omar, Riya Shah, Angela Colantonio

**Affiliations:** 1 KITE-Toronto Rehabilitation Institute, University Health Network, Toronto, Ontario, Canada; 2 Institute of Health Policy, Management and Evaluation, University of Toronto, Toronto, Ontario, Canada; 3 Rehabilitation Sciences Institute, University of Toronto, Toronto, Ontario, Canada; 4 Department of Health and Society, University of Toronto Scarborough, Scarborough, Ontario, Canada; 5 Department of Psychology, University of Toronto Scarborough, Scarborough, Ontario, Canada; 6 Department of Occupational Science & Occupational Therapy, University of Toronto, Toronto, Ontario, Canada; 7 Dalla Lana School of Public Health, University of Toronto, Toronto, Ontario, Canada; Emory University, UNITED STATES

## Abstract

Traumatic brain injuries (TBI) and mental health or substance use disorders (MHSU) are global public health concerns due to their prevalence and impact on individuals and societies. However, care for individuals with TBI and MHSU remains fragmented with a lack of appropriate services and supports across the continuum of healthcare. This systematic review provided an evidence-based foundation to inform opportunities to mobilize and adapt existing resources to integrate care for individuals with TBI and MHSU by comprehensively summarizing existing integrated activities and reported barriers and facilitators to care integration. MEDLINE, EMBASE, PsycINFO, CINAHL, Cochrane Central Register of Controlled Trials, Sociological Abstracts, and Dissertations & Theses Global were independently reviewed by two reviewers based on pre-determined eligibility criteria. Data on the integration activity, level and type of integration, reported barriers and facilitators, and the strategies aligning with the World Health Organization’s (WHO) Framework on Integrated Person-Centred Care were extracted to form the basis for a narrative synthesis. Fifty-nine peer-reviewed articles were included, describing treatments (N = 49), programs (N = 4), or screening activities (N = 7). Studies discussing clinical integration at the micro- (N = 38) and meso- (N = 10) levels, service integration at the micro- (N = 6) and meso- (N = 5) levels, and functional integration at the meso-level (N = 1) were identified. A minority of articles reported on facilitators (e.g., cognitive accommodations in treatment plans; N = 7), barriers (e.g., lack of education on cognitive challenges associated with TBI; N = 2), or both (N = 6), related to integrating care. This review demonstrated that integrated TBI and MHSU care already exists across a range of levels and types. Given the finite and competing demands for healthcare resources, cognitive accommodations across treatment plans to facilitate integrated TBI and MHSU care should be considered. Multidisciplinary teams should also be explored to provide opportunities for education among health professionals so they can be familiar with TBI and MHSU.

**Trial registration:** Prospero Registration: CRD42018108343.

## Introduction

Traumatic brain injuries (TBI), mental health disorders, and substance use are global public health concerns due to their prevalence and impact on individuals and societies [1–4]. TBI has been defined as an alteration in brain function, or other evidence of brain pathology, caused by an external force [5]. Estimates suggest that 50% of the world’s population will sustain a TBI in their lifetime, costing the global economy $400 billion USD per year [2]. Mental health and/or substance use disorders (MHSU) are a range of disorders characterized by changes in emotions, behaviours, and thinking [6]. As of 2016, MHSU was estimated to affect 16% of the world’s population [7]. The 2010 Global Burden of Diseases Study documented that MHSU is the leading cause of non-fatal burden of disease, measured by years of life lived with disability, and accounts for 23% of all non-fatal burden, globally [4]. By the year 2030, MHSU alone is expected to cost $6.0 trillion worldwide [8].

While TBI and MHSU are significant concerns independently, many individuals with TBI also experience MHSU [9–12] and many individuals with MHSU have also suffered a TBI [12–14]. For example, an assessment of 295 individuals receiving treatment for MHSU found 80% of participants screened positive for a TBI [14]. Population-level surveys have found a significant association between a lifetime history of TBI and elevated psychological distress, cannabis use, suicidal ideations, and attempted suicides [9, 10]. A prospective longitudinal cohort study found that at 3 and 6 months post-TBI, almost 20% of patients experienced post-traumatic stress disorder (PTSD), and nearly 10% of patients experienced depression [11]. Similarly, surveillance data on adults with a history of TBI with loss of consciousness demonstrated that they had 2.1 times higher odds of lifetime depression and 1.7 times higher odds of binge drinking [12]. Other common types of MHSU among individuals with TBI include anxiety, obsessive-compulsive disorder, attention deficit hyperactivity disorder, and schizophrenia [15–19]. Furthermore, the symptoms of TBI and MHSU can overlap and exacerbate one-another [19–22]. For example, depression can exacerbate physical symptoms of TBI such as headaches, pain, and fatigue and these physical symptoms can exacerbate depression if left untreated [23]. Treating one without acknowledging and accounting for the impacts of the other can lead to negative health outcomes for both TBI and MHSU. The interconnectedness of TBI and MHSU symptoms also necessitate adjustments to treatments of one or the other when the conditions are comorbid. For example, treatments for PTSD may need to be adjusted to accommodate for the potential difficulties with emotion regulation, impulse control, pain, and cognitive limitations that may accompany a TBI [21]. Unfortunately, healthcare for individuals with TBI and MHSU remains fragmented, with a lack of appropriate services and supports across the continuum of healthcare [24–26].

Integrated healthcare centred on the needs of the individuals, their families, and communities has been acknowledged by the World Health Organization (WHO) as a solution to address multi-morbidities and improve access to care and patient outcomes [27]. In 2016, the WHO published the “Framework on Integrated People-Centred Health Services” (hereafter referred to as the ‘WHO Framework’). It defines integrated health services as:

Health services that are managed and delivered so that people receive a continuum of health promotion, disease prevention, diagnosis, treatment, disease-management, rehabilitation and palliative care services, coordinated across the different levels and sites of care within and beyond the health sector, and according to their needs throughout the life course [27 p.2].

Crucially, it is widely acknowledged that health system or health service integration is highly complex and includes a range of strategies [28–32] that can be distinguished by, for example, the type (organizational, functional, service, clinical) or level (micro, meso, macro) of integration [28]. Indeed, existing reviews on integrating services across health conditions such as human immunodeficiency virus (HIV), cardiovascular diseases, substance use, and cancer [33–38], have identified integrated activities ranging from meso-level clinical integration in the form of screening for substance use or cardiovascular disease at HIV facilities to micro-level service integration in the form of pharmacological treatment for HIV at a substance use facility [33, 34].

To the best of our knowledge, there is currently no systematic review on integrated care for individuals with TBI and MHSU, despite the prevalence of their co-occurrence, the interrelatedness of their symptoms, and the special considerations needed when they are comorbid. This is a significant gap in informing opportunities to integrate TBI and MHSU care. This systematic review aimed to summarize the current levels and types of integrated care for TBI and MHSU and identify the reported barriers and facilitators to integrated care for TBI and MHSU. Findings from this review provide an evidence-based foundation to inform opportunities to integrate person-centred healthcare for individuals with TBI and MHSU.

## Methods

The protocol for this systematic review is published in the journal *BMJ Open* [39] and is highlighted below.

### Search strategy

The following databases were first searched in August 2018 for relevant articles. A second search was conducted in September 2020 to retrieve articles indexed since August 2018:

1. MEDLINE In-Process and Other Non-Indexed Citations and MEDLINE
2. EMBASE
3. PsycINFO
4. CINAHL
5. Cochrane Central Register of Controlled Trials
6. Sociological Abstracts
7. Dissertations & These Global.

The search strategy was informed by published protocols and reviews on integrated healthcare [33–38, 40] and the WHO Framework [27]. Text words and subject headings (e.g., Medical Subject Headings [MeSH], Emtree) related to the following concepts were used: (a) TBI, (b) MHSU, (c) integrated care, (d) barriers and facilitators, and (e) healthcare access. The primary search strategy comprised of concepts (a) + (b) + (c) to capture papers that discuss integrated care for TBI and MHSU. A secondary search strategy, comprised of (a) + (b) + (d) + (e), was conducted to capture papers that discuss barriers and facilitators to accessing TBI and MHSU care. These two searches were combined with an ‘OR’ statement to complete the search strategy for each database. The search strategy for Sociological Abstracts was reduced to concept (a) + (b) due to paucity of results. Where possible, searches were limited to English language and excluded animal studies, conference abstracts, magazines, books, and encyclopedias. EndNote and Covidence [41] were used to manage all the records returned from this search strategy. **S1 File** presents the search strategy associated with each database.

### Study selection

Peer-reviewed qualitative, quantitative, and mixed methods articles, as well as dissertations identified through our original search or through reference lists of included primary research articles and scoping or systematic reviews that met the following inclusion criteria were included in this systematic review:

1. Describe or evaluate a policy, program, or intervention/treatment at the health service delivery level for individuals with TBI and MHSU; OR
2. Screen or diagnosis for TBI in a mental health or substance use service setting; OR
3. Screen or diagnosis for MHSU in a brain injury health service setting.

Narrative or commentary articles, those that describe a theory or framework without primary data reported, and articles that examine a broader brain injured population (e.g., acquired brain injury, trauma patients) without specific mention of TBI were excluded. Furthermore, only articles published in or after 2013 were included in this review. It is acknowledged that integrated care predates the start date of our review; however, several factors informed our decision to only include articles published in 2013 or later. Almost 50% of the title and abstracts returned from this review’s search strategy were published in or after 2013 and a PubMed search of “traumatic brain injury” AND (“mental health” OR “substance use”) showed an almost 100% increase in publications from 2013 to 2018. This suggests an increase in recognition and treatment of TBI in recent years as well as the growing acknowledgement of MHSU as a symptom or comorbidity of TBI. Thus, the inclusion of articles published in or after 2013 also captures more recent health service trends and provides a more contemporary review. However, we acknowledge that in limiting the search in this way we have missed relevant articles published prior to 2013.

All articles were independently assessed by at least two reviewers (VC, DT, SO). At the title and abstract screen, articles were considered for the full-text screen if they described or evaluated a policy, program, or intervention/treatment at the health service delivery level for individuals with TBI or MHSU. Consistent with the peer-reviewed protocol [39], a random sample of articles at the title/abstract and full-text screening stages were screened by at least two reviewers who then met to discuss and reach consensus. This consensus then served as a guide to clarify the eligibility criteria and to ensure consistency. Throughout the screening process, all reviewers met regularly to review the screened articles, discussed any challenges with applying the eligibility criteria, and consulted with a third reviewer (VC, DT, or SO) to resolve discrepancies when needed.

### Data extraction and synthesis

A narrative synthesis, to critically analyze and report the peer-reviewed literature [42], was conducted using data extracted independently by one reviewer (VC or DT) and peer-reviewed by a second reviewer (VC, DT, SO, or RS). The extracted data were tabulated and stratified by sex or gender where possible. Studies reporting on men and women were noted as capturing gender while studies reporting on males and females were noted as capturing sex. Each integration activity was grouped by the type of activity (policy, diagnosis/screening, or treatment/intervention), level and type of integration [28], and the five recommended strategies for integrated care using the WHO Framework [27], defined in **Table 1**. As dissertations have not been formally peer-reviewed, they were not included in the narrative synthesis but described separately to capture early work, such as pilot studies, of integrated care that may not yet be published in peer-reviewed journals.

**Table 1:** Definitions.

**Level of Integration [28, 32]**	
Micro	Integration at the individual level, in which providers deliver integrated care for individuals through coordination of care, care planning, use of technology, or other approaches.
Meso	Integration at the sub-group or sub-population level, in which providers deliver integrated care for groups of individuals or populations with the same disease through care pathways or other approaches.
Macro	Integration at the population level, in which providers deliver integrated care to populations they serve.
**Type of Integration [28]**	
Clinical	Integration of care delivered by professionals and providing to patients into a single or coherent process within and/or across professionals.
Service	Integration of different clinical services at an organizational level.
Functional	Integration of non-clinical support and back-office functions.
Organizational	Integration of organizations through mergers or ‘collectives’ and/or virtually through coordinated provider networks.
**WHO’s Five Strategies for Integrated People-Centred Health Services [27]**	
1. Engaging and empowering people and communities	1.1 Engaging and empowering individuals and family 1.2 Engaging and empowering communities 1.3 Engaging and empowering informal carers 1.4 Reaching the underserved and marginalized
2. Strengthening governance and accountability	2.1 Bolstering participatory governance 2.2 Enhancing mutual accountability
3. Re-orienting the model of care	3.1 Defining service priorities based on life-course needs, respecting social preferences 3.2 Revaluing promotion, prevention, and public health 3.3 Building strong primary care-based systems 3.4 Shifting towards more outpatient and ambulatory care 3.5 Innovating and incorporating new technologies
4. Coordinating services within and across sectors	4.1 Coordinating care for individuals 4.2 Coordinating health programs and providers 4.3 Coordinating across sectors
5. Creating an enabling environment	5.1 Strengthening leadership and management for change 5.2 Strengthening information systems and knowledge 5.3 Striving for quality improvement and safety 5.4 Reorienting the health workforce 5.5 Aligning regulatory frameworks 5.6 Improving funding and reforming payment systems

### Quality assessment

All articles meeting the inclusion criteria were assessed for risk of biases using quality assessment tools designed by methodologists from the National Institutes of Health and Research Triangle Institute International [43] or the Critical Appraisal Skills Program checklist [44]. One reviewer independently assessed the quality of all included studies (VC or SO), and they were peer-reviewed by a second reviewer (RS or VC). These tools were designed to assist reviewers in assessing risk of biases and were not intended to generate a score to be used to arrive at quality assessment [43]. As such, the risk of biases associated with each article was recognized during the narrative synthesis and discussion of this review. No articles were excluded from the review or narrative analysis based on their risk of bias.

## Results

**Fig 1:**
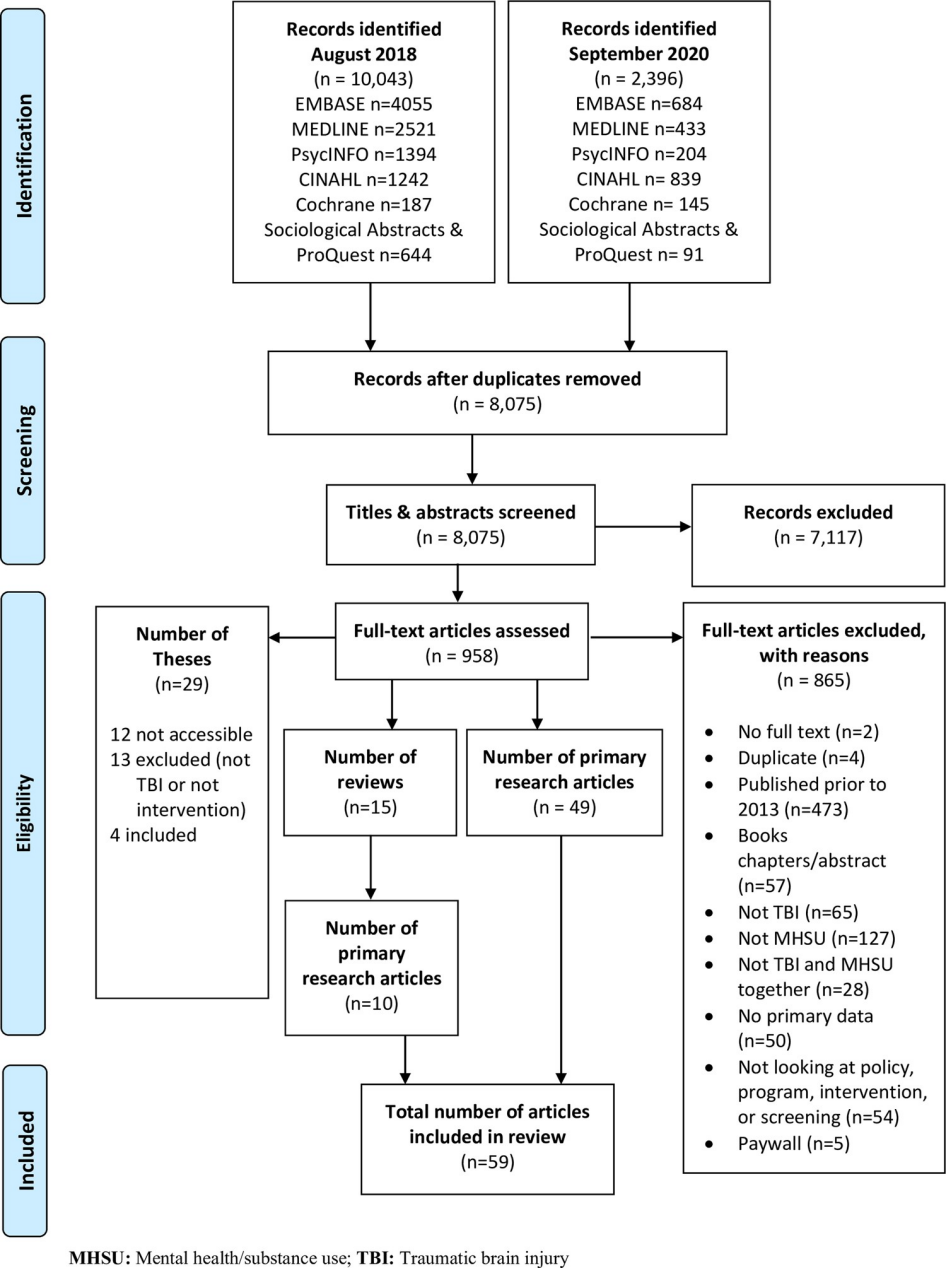
PRISMA flow chart.

A total of 12,439 articles were identified through the database searches. After duplicates were removed, 8,075 title and abstracts were screened, of which 958 were identified for full text review. Among these, 473 articles were published prior to 2013 and 57 were book chapters/abstracts and were excluded from further review. Of the remaining full text articles reviewed, four dissertations [45–48] and 59 research articles (49 identified through the search and an additional 10 identified from 15 reviews that met inclusion criteria), were included in this review [49–107]. **Fig 1** presents the Preferred Reporting Items for Systematic Reviews and Meta Analyses (PRISMA) flow diagram for a detailed account of the search and screening results.

### Peer-reviewed findings

Peer-reviewed articles were primarily conducted in the United States (81%). Thirty one percent of studies included all severity-levels of TBI; 22% did not specify TBI severity. PTSD and depression were the most commonly investigated MHSU (56% and 46%, respectively). Many of the included studies (51%) focused on veterans, all of which were conducted in the United States. Except for case studies, which predominantly included a single sex or gender of participant, none of the included studies stratified results based on sex or gender. The most common types of integration activities were treatments/interventions (N = 49). Four studies reported on program implementation and seven studies described screening activities. One study described both screening and treatment activities, however, the type of treatment was not detailed in the study [50]. **Table 2** presents study characteristics and integration activities of the included peer-reviewed studies with study citations. **S1 Table** presents key data extracted from these articles.

**Table 2:** Study characteristics and description of integration activities.

**Study Characteristics**	**N**
**Country of Study**	
United States [49, 50, 53–57, 59–72, 74–76, 78–87, 89–91, 93–95, 98–102, 104–106]	48
Australia [73, 92, 103]	3
China [88, 107]	2
South Africa [51]	1
Canada [58]	1
India [52]	1
Israel [96]	1
Switzerland [77]	1
United Kingdom [97]	1
^a^ **Study Design**	
Controlled interventions [52, 54, 58, 59, 66, 67, 70–72, 74, 76, 79, 82, 84, 90, 92, 95, 100, 103, 104, 107]	21
Case studies/series [49, 53, 56, 57, 60, 68, 73, 77, 78, 81, 89, 96, 97, 99, 101, 102, 106]	17
Observational cohort/cross-sectional [51, 61, 62, 75, 85, 88, 91, 93, 94, 98, 100]	11
Before-after no control groups [63–65, 69, 80, 83, 86, 105]	8
Qualitative [50, 67, 87]	3
Case-control [55]	1
**Age (Years)**	
Adolescents, adults, or older adults (≥16) [53, 75, 88, 92]	4
Adults only (18–64) [49, 51, 52, 54–56, 58–60, 62–74, 76–81, 83–87, 89–91, 93–103, 105–107]	50
Adults or older adults (18+) [61, 82]	2
Older adult (90) [57]	1
Not reported [50, 104]	2
**Sex/Gender**	
^b^ Data by sex or gender [73, 81, 101, 106]	4
^c^ Males/men only [52, 63, 64, 67, 90]	5
**Veterans Only**	
^d^ Yes [55, 56, 60, 61, 63–65, 67–70, 74, 79–81, 84–87, 90, 91, 93, 94, 98, 100–102, 104–106]	30
**TBI Severity**	
All [50, 51, 54, 59, 61, 66, 69, 72, 74, 76, 82, 83, 85, 86, 88, 92, 104, 105]	18
Mild [53, 55, 56, 60, 65, 67, 75, 80, 81, 87, 90, 100]	12
Moderate [62, 97, 102]	3
Severe [49, 77, 78, 103]	4
Mild and moderate [52, 71, 79, 84, 95, 107]	6
Mild and severe [73]	1
Moderate and severe [70, 101]	2
NR [57, 58, 63, 64, 68, 89, 91, 93, 94, 96, 98, 99, 106]	13
^e^ **MHSU**	
PTSD [50, 51, 55, 56, 60, 62–65, 67–70, 74, 75, 79–81, 84–87, 89, 90, 93, 94, 97, 100–102, 104–106]	33
Depression [49, 50, 52–54, 57–59, 62, 66, 67, 71–73, 76, 78, 82, 83, 88–90, 92, 95, 99, 103, 104, 107]	27
Anxiety [50, 51, 53, 62, 67, 76, 83, 90, 92, 96, 99, 103]	12
Substance use [68, 74, 90]	3
Obsessive compulsive disorder [77, 90]	2
Attention-deficit hyperactive disorder [49]	1
Not reported (all sought mental health services) [61, 91, 98]	3
**Description of Integration Activity**	**N**
^f^ **Treatment/Intervention**	**49**
Psychotherapy [53, 54, 58–60, 62, 65–67, 69, 70, 72–74, 76, 77, 79, 81, 84–86, 88, 90, 92–94, 97, 100–103, 105]	32
Pharmacotherapy [52, 56, 71, 77, 82, 88, 96, 107]	11
rTMS [78, 95, 99]	3
Vestibular rehabilitation [53, 63, 64]	3
Hypnotic induction [68, 89]	2
Virtual reality [87]	1
Alternative medicine [96]	1
Animal therapy [106]	1
Psychosocial interventions [57]	1
**Program**	**4**
Psychotherapy and pharmacotherapy provided by health professionals across mental health and brain injury clinics [49]	1
Psychotherapy and pharmacotherapy (to address TBI and MHSU) and vestibular rehabilitation and musculoskeletal therapy (to address sequelae of TBI) [80]	1
Embedded psychologist and psychiatrist within interdisciplinary team [55]	1
In-home assessment, intervention, and home modification by OT [104]	1
**Screening**	**7**
Screening for TBI within MHSU setting [61, 90, 98]	3
Screening for MHSU within brain injury clinics, EDs, and adult trauma centre [50]	1
Screening for MHSU within ED [75]	1
Screening for MHSU within hospital [51]	1
Screening for MHSU within community setting [83]	1

Notes

^a^ Two research articles [67, 100] reported two studies of different study designs and as such, data reported on study design are for 61 studies

^b^ All case studies

^c^ Excludes case studies; two before-after without control group, three controlled interventions

^d^ All conducted in the United States

^e^ Sixteen studies [49–51, 53, 62, 67, 68, 74, 76, 83, 89, 90, 92, 99, 103, 104] examined integrated care for individuals with TBI and more than one type of MHSU

^f^ Some studies include more than one type of treatment and as such, the number of articles reported across treatment types exceeds 49 studies

**ED:** Emergency department; **MHSU:** Mental health disorders or substance use; **OT:** Occupational therapy; **PTSD:** Post-traumatic stress disorder; **rTMS:** Repetitive transcranial magnetic stimulation; **TBI:** Traumatic brain injury

Risk of bias assessment was conducted for studies that reported a treatment or program (N = 52, two studies incorporated two study designs): 21 randomized controlled trials (RCTs), 17 case studies/series studies, 7 before-after studies without a control, 6 observational cohort or cross-sectional studies, 2 qualitative studies, and 1 case-control study (**S1 Table**). Risk of bias associated with selection (N = 48), information (N = 41), performance (N = 33), and reporting (N = 17) were common due to the diverse study designs included in this review. As the aim of this review was to summarize existing integrated TBI and MHSU care rather than evaluate the outcome of the integrated activities, no studies were excluded based on their risk of bias. However, these biases were recognized in the narrative synthesis and discussion of the review.

#### Level and type of integration

The majority of the includes studies discussed integration at the level of professionals providing care (i.e., clinical integration) at the level of individuals (i.e., micro level integration) (N = 38) or at the meso or sub-group/sub-population level (N = 10). Several studies discussed integration of different clinical services (i.e., service integration) at the micro (N = 6) and meso level (N = 5). One study described meso-level care that was both service and functional integration [55]. None of the included studies discussed integration at the macro or population/organization level. **Table 3** summarizes the levels and types of integrated care as defined in **Table 1**.

**Table 3:** Summary of levels and types of integrated care.

Levels and Types of Integrated Care	N
**Micro Level–Clinical Integration**	**38**
Psychotherapy delivered by health professionals to patients who met eligibility criteria to:	26
a) Address psychosis [53], depression [54, 58, 59, 66, 72, 92, 103], anxiety [92], TBI [62], or sequelae of TBI and/or MHSU [60, 65, 69, 70, 105]	
b) Improve participation in treatments, mental health, and life management [73, 76, 84, 97]	
c) Understand the impact of TBI on psychotherapy for individuals with PTSD [93] or PTSD and SUD [74]	
Pharmacotherapy to treat depression [52, 71, 82] or depression and other sequela after TBI [107]	4
rTMS to treat depression [78, 95] or neuropsychiatric sequelae [99] of TBI	3
Vestibular rehabilitation to address PTSD symptoms among veterans who suffered combat-related TBI [63, 64]	2
Case manager who coordinated care and provide psychosocial intervention [57]	1
Hypnotic inductions [68] or animal therapy [106] to treat PTSD among Veterans who suffered combat-related TBI	2
**Micro Level–Service Integration**	**6**
Pharmacotherapy and psychotherapy to address OCD [77] and depression [88] after TBI	2
Pharmacotherapy, acupuncture, and herbal formula to address PTSD among an individual with TBI integrating pharmacological therapy [96]	1
Integration of a variety of treatments (e.g., hypnosis, aqua therapy, acupuncture, spiritual counseling) to address anxiety and depression among an individual with TBI [89]	1
Psychotherapy and vestibular therapy to treat psychotic symptoms after TBI [53]	1
Program integrating multidisciplinary teams (OT, PT, SLP, psychologist, neuropsychologist, medical specialists) to provide psychotherapy and pharmacotherapy to address PTSD, anxiety, and depression and vestibular rehabilitation and musculoskeletal therapy to address other symptoms associated with TBI [80]	1
**Meso Level–Clinical Integration**	**10**
Screening for TBI within MHSU settings [61, 91, 98]	3
Screening for MHSU among patients with TBI in:	3
a) general hospital setting [51]	
b) ED setting [75]	
c) Brain injury clinics, EDs, and adult trauma centres [50]	
Screening for MHSU among community-based adults with TBI using a smartphone application [83]	1
Art therapy introduced in outpatient medical facility to address PTSD and TBI symptoms [85]	1
Virtual reality grocery store for therapists working with veterans with PTSD or mTBI for assessment and intervention of cognitive impairments and emotional challenges associated with mTBI and PTSD [87]	1
Home-based, family-inclusive program for veterans with TBI at a polytrauma program, where an OT meets with veterans and family members in their homes to identify goals, develop an action/treatment plan, and introduces home modifications [104]	1
**Meso Level–Service Integration**	**5**
Integration of neuropsychiatrists (to provide medication) and behavioural therapists (to provide psychotherapy) to address PTSD among individuals with mTBI at polytrauma rehabilitation centre [56]	1
Acceptance and Commitment Therapy workshop developed by clinical psychologists, neuropsychiatrists, cognitive psychologists, chaplains, and anthropologists for veterans from medical centres, community-based outpatient clinics, and local community locations [67]	1
Embedded cognitive rehabilitation intervention to standard vocational rehabilitation to manage cognitive symptoms and negative emotions for individuals with TBI and MHSU [90]	1
Integrated team-based care at a post-acute outpatient rehabilitation for veterans with TBI by integrating neuropsychiatrists (to provide medication) and behavioural therapists (to provide trauma-focused therapy); active mental health issues were discussed at a formal Mental Health Interdisciplinary Treatment Team meeting or informal in-person discussion [55]	1
Program of Enhanced Psychiatrist Services, a brain injury outpatient psychiatric program that provided psychotherapy from mental health therapists, pharmacotherapy treatment from neuropsychiatrist, and group therapy co-led by therapists from the psychiatric and brain injury clinics; multidisciplinary team meetings were also held [49]	1
**Meso Level–Functional Integration**	**1**
Integrated team-based care where electronic health records were also used for communications; for example, to alert psychiatrist to a mental health consult and schedule an initial evaluation, to resolve issues or concerns such as those related to polypharmacy, receipt of care in timely manner, or delay in rehabilitation due to unstable mental health condition [55]	** **
	1
	** **
	** **

**ED:** Emergency department; **MHSU:** Mental health disorders or substance use; **mTBI:** Mild traumatic brain injury; **OCD:** Obsessive compulsive disorder; **OT:** Occupational therapist; **PT:** Physiotherapist; **TSD:** Post-traumatic stress disorder; **rTMS:** Repetitive transcranial magnetic stimulation; **SLP:** Speech language pathologist; **SUD:** Substance use disorder; **TBI:** Traumatic brain injury

#### Strategies for integrated person-centred healthcare

Almost all included studies (N = 51) described activities that involved engaging and empowering individuals and families (i.e., WHO Framework Strategy 1.1). Coordinating services, aligning with WHO Framework Strategy 4, was the second most commonly described integration strategy, with six studies focusing on screening for TBI in MHSU settings or vice versa and 12 studies focused on coordination for individuals, one of which also considered coordination across sectors. Studies focusing on re-orienting the model of care (i.e., Strategy 3), solely aligned with the strategy of innovating and/or incorporating new technologies (N = 7). **Table 4** presents more detailed reporting on alignment between WHO Framework Strategies and included articles.

**Table 4:** Summary of WHO Framework strategies addressed by included studies.

WHO Framework Strategies [27]		N = 59^a^
1. Engaging and empowering people and communities	1.1 Engaging and empowering individuals and family [49, 52–60, 62–74, 76–81, 84–90, 92–97, 99, 101–107]	51
3. Re-orienting the model of care	3.5 Innovating and incorporating new technologies [70, 73, 76, 83, 87, 90, 93]	7
4. Coordinating services within and across sectors	4.1 Coordinating care for individuals [49, 53, 55, 57, 67, 77, 80, 88–90, 96, 104] 4.3 Coordinating across sectors [96] Screening (broadly strategy 4) [50, 51, 61, 75, 91, 98]	18

Note

^a^ Some studies addressed more than one strategy, so the number of articles reported across strategies exceeds 59 articles

#### Barriers and facilitators

A minority of studies (N = 15) reported on barriers or facilitators, approximately half of which solely discussed facilitators (N = 7). The remaining eight articles either discussed both barriers and facilitators (N = 6) or just barriers (N = 2). Common reported barriers included lack of education, limited access to care, and difficulties using or navigating technology. Commonly reported facilitators included engaging patients and families or caregivers, collaborating across disciplines, and incorporating compensatory strategies for addressing TBI-related cognitive challenges into treatment plans. **Table 5** presents barriers and facilitators reported in the articles. An additional nine studies did not explicitly report barriers and facilitators to treatment activities but noted that compensatory strategies to address cognitive challenges associated with TBI were incorporated in the treatment design [54, 58–60, 66, 72, 79, 84, 92].

**Table 5:** Summary of barriers and facilitators.

Description of barriers and facilitators	N
**Barrier**	**8**
Lack of education among caregiver, patients, and healthcare professionals on MHSU and TBI symptoms [50]	1
Limited access to care due to:	
Geography of care facilities [49]	1
Financial limitations (cost of care/lack of insurance) [50, 96]	2
Hesitancy of healthcare providers to diagnose without relevant experience [50]	1
Difficulties in using/navigating technology (virtual reality, computers/laptops, mobile apps) [87, 90]	2
Assessment questions:	
Complex language [83]	1
Different measurement scales and timeframe references in same assessment [90]	1
Cognitive challenges (e.g., forgetting skills, misplacing materials) [67]	1
Polypharmacy [50]	1
Cultural differences:	
Language resulting in different understanding of questions [51]	1
Defining MHSU questions [51]	1
**Facilitator**	**13**
Inclusion of family or caregivers in treatment process [49–51, 104]	4
Compensatory strategies to address cognitive challenges associated with TBI [65, 67, 101, 105]	4
Education for patients and healthcare professionals regarding symptoms and outcomes associated with TBI and MHSU [49, 101]	2
Knowledge of care pathways [57]	1
For studies leveraging technology: simple navigations and clear content and questions [83, 90]	2
Collaboration between individuals and health professionals across disciplines [55, 57]	2
Patient advocacy and empowerment [57, 67, 81]	3
Co-location of treatments to improve access and engagement [55]	1
Interpreter [51]	1

**MHSU:** Mental health disorder or substance use; **TBI:** Traumatic brain injury

### Dissertation findings

The four dissertations that met inclusion criteria were all completed at American Universities. One specifically focused on mild TBI [48], with the remainder not specifying a severity [45–47] and two focused on veterans [46, 47]. PTSD [48] and depression [45] were each the focus of one dissertation. One specifically reported on females [45], but the remainder did not stratify results by sex or gender. Two dissertations reported treatments, one a cognitive behavioural therapy group designed to address depression symptoms while accommodating the cognitive needs of women with TBI [45] and the other a therapeutic horseback riding intervention for veterans [46]. Both are examples of micro level clinical integrations. The other two dissertations reported on screening, one for PTSD in hospitalized trauma patients [48] and the other a computerized screening assessment for MHSU among veterans [47]. Both are examples of meso level clinical integrations.

## Discussion

This review is the first, to the best of our knowledge, to summarize existing levels and types of integrated care for individuals with TBI and MHSU and, where reported, the barriers and facilitators to integrated care. Fifty-nine peer-reviewed articles and four dissertations published in 2013 or later were identified that met our inclusion criteria. The majority of peer-reviewed articles and all dissertations discussed micro- or meso-level clinical integrations and a minority of peer-reviewed studies reported on service integration at these levels. None of the included studies discussed integrations at the macro-level or the level of populations or organizations, which may be reflected in grey literature, including government organization reports, rather than the peer-reviewed literature. Integration strategies from included peer-reviewed articles were mapped onto the WHO’s five strategies for integrated people-centred health services [27]. Almost all studies noted engaging and empowering individuals and their families as a critical element to person-centred integrated care, aligned with the WHO Framework strategy 1. Coordination of care, aligned with strategy 4, was noted among almost a third of the included studies. Re-orienting the model of care, specifically through innovation and incorporating new technologies, was also discussed in a minority of studies. Notably, none of the included studies discussed strategy 2, strengthening governance and accountability, or strategy 5, creating an enabling environment. This is perhaps due to the dearth of studies reporting on macro level integration. Only a small subset of articles reported on barriers or facilitators, with barriers including lack of education, limited access, and difficulties with technology, and facilitators including, patient and family engagement, collaboration among care providers, and compensatory strategies for cognitive challenges. It is notable that an additional subset did not explicitly discuss barriers and facilitators but highlighted the use of compensatory strategies to accommodate TBI-related cognitive challenges. Below, we further discuss some of the key findings and highlight considerations for integrated TBI and MHSU care and future research directions in this field as identified through the narrative synthesis.

Like other reviews on integrated care [33–38, 40], the integration of TBI and MHSU care already exists at the micro-level in the form of treatments or interventions delivered by a healthcare professional. Studies included in this review also indicated some integrated care occurring at the meso-level, predominantly in the form of screening for TBI among individuals with MHSU or vice versa [50, 51, 61, 75, 83, 91, 98] and multidisciplinary care teams [49, 55, 56, 67, 90]. These meso-level studies were predominantly single program initiatives working on a relatively small scale. As previously noted, none of the included studies discussed integrated care at the macro-level. This lack of literature could be because no population-level integrated care initiatives exist for this population or because no peer-reviewed evaluation or analysis of these initiatives have taken place. The lack of peer-reviewed research on population-level integration may be due to financial and human resources associated data collection at a population-level, particularly in settings without population-based data that may be collected as part of a publicly funded health system. Nonetheless, macro-level integrations may be discussed in government or organization reports rather than in the peer reviewed literature. Without a grey literature search, we cannot comment on the presence or absence of macro-level integrations for TBI and MHSU. Further investigation into the reason for a lack of studies and efforts to contribute evidence to this body of literature is warranted to inform how integrated care might be implemented, including barriers and facilitators to integrated care, or how effective that care might be for individuals with TBI and MHSU at the population level. Integrated healthcare centred on the needs of the individuals, their families, and communities has been acknowledged by the World Health Organization (WHO) as a solution to address multi-morbidities and improve access to care and patient outcomes [27]. As such, health administrators and decision-makers are encouraged to recognize comorbid health conditions in planning and implementing integrated care.

Notably missing from the articles reporting on screening activities was any description of the outcome of the screening such as access to care or receipt of treatment. All screening studies included in this review aimed to identify the prevalence of TBI or MHSU or assess the feasibility of select tools to screen for TBI or MHSU. While screening and the receipt of a diagnosis may provide opportunities to access treatments, MHSU diagnosis may also be a barrier to TBI treatment. Indeed, some RCTs included in this review had exclusion criteria based on types of MHSU or severity of TBI [52, 54, 58, 59, 66, 67, 71, 72, 74, 76, 79, 82, 84, 90, 95, 103, 107]. As such, potential negative consequences of TBI and/or MHSU diagnosis should be considered when implementing screening as a form of integrated care. In line with the WHO Framework, the engagement of individuals with lived experience of TBI and MHSU is critical when considering screening activities, to mitigate negative, unintended effects.

Though only a subset of articles reported on barriers and facilitators, it is worth highlighting that many of those noted were complementary. For example, one study noted the geographical distance between care facilities as a barrier [49] and another noted co-location of treatments was a facilitator to improving access [55]. The most commonly reported facilitator was compensatory strategies for cognitive challenges. Thirteen of the included studies specifically noted accommodations in treatment to address cognitive challenges associated with TBI, four studies listed these as accommodations as facilitators [65, 67, 101, 105], while an additional nine studies simply noted that the accommodations were put in place to support participants [54, 58–60, 66, 72, 79, 84, 92]. The accommodations reported were aimed at overcoming barriers in participation, improving treatment adherence, and supporting task completion and ranged from appointment reminders [65], to psychoeducation [101], to adjusting the frequency and duration of sessions [67, 101]. One study noted unaddressed cognitive challenges, specifically memory-related challenges with remembering content and keeping track of materials, as a barrier to care provision [67]. Taken together, these studies report on a range of strategies to address cognitive challenges. The value of cognitive accommodations for TBI has also been recognized outside of the research included in this review [21, 108–110]. For example, accommodations for cognitive, emotional, and physical challenges related to TBI may improve the effectiveness of PTSD treatment for individuals with comorbid TBI and PTSD, particularly when they have been deemed “non-responsive” to treatments normally deemed effective [21]. Therefore, reviews on the effectiveness of incorporating accommodations for cognitive challenges across the treatment plan as a way of accommodating individuals who may have TBI are encouraged.

While this review’s goal was not to assess the efficacy of integration activities for individuals with TBI and MHSU (and therefore, not systematically summarized in this review), we did see a trend among included studies that we believe is worth noting. Specifically, almost all studies, regardless of integration activity, level, or type, reported positive outcomes. We recognize that this finding may be impacted by reporting and publication bias. Nonetheless, this supports the widely accepted notion that there is no ‘one size fits all’ approach to integrated care [30, 31]. Clinical integrations identified in this review reported improved outcomes (e.g., reduced symptoms, improved participation and outcomes), indicating that the positive impact of integrated TBI and MHSU care can be achieved without integration across teams or disciplines. However, service integration, particularly meso-level integrations, may provide added benefits to the individuals, their families or caregivers, and health professionals by addressing some barriers identified in this review. For example, the inclusion of diverse disciplines in the treatment plan to address cognitive and motor impairments may be beneficial [80] as the challenges associated with cognitive and motor impairments reduce functional gain during inpatient rehabilitation among individuals with TBI [13]. For families and caregivers, collaboration between disciplines at a single facility may reduce travel-related barriers, as traveling between facilities, often in the same day, has been noted as a barrier for accessing required care from different providers [49]. Finally, team-based care or, at the very least, informal multidisciplinary team meetings and discussions may offer opportunities for education on TBI and MHSU [49, 55]. This is particularly important because the lack of experience with TBI and MHSU was a noted barrier to diagnosis, contributing to delayed treatment [50]. Future reviews focused on investigating the efficacy of integrated care is encouraged to inform integrated care for individuals with TBI and MHSU.

Finally, even though most studies aligned with the WHO Framework strategic approaches ‘Empowering and engaging people and communities’, none of the articles included in this review specifically addressed the needs of underserved or marginalized populations experiencing TBI and MHSU. The WHO Framework defines marginalized populations as comprising individuals who may not receive or access care due to barriers including but not limited to income, education, race/ethnicity, and gender [27]. We acknowledge that the lack of articles that engage underserved and marginalized population may be reflective of our search strategy, as the search did not specifically include search terms to identify these populations. However, the intersectional nature of health inequities highlight the importance of focusing on health disparities that disproportionately impact marginalized populations. Addressing integrated care involves considering the health inequities resulting from unequal social relations according to gender inequity, racism, and social and economic exclusion, which disproportionately impact underserved populations [111]. As noted by strategic approach 1.4, programs for underserved or marginalized populations must be available to address health equity and guarantee universal access to quality healthcare. Additional work focusing on the experiences of underserved, marginalized, or racialized communities, such as the recent critical transdisciplinary scoping review mapping integrated care pathways for Black people experiencing TBI [112], are needed to ensure integrated care adequately and effectively addresses the needs of these communities.

Similarly, while some studies included in this review successfully leveraged or incorporated technology (e.g., virtual reality-assisted treatment, mobile applications) in the integrated activities, there was little consideration of the barriers related to technology use among underserved populations. Developers must be mindful of potential challenges associated with access to and use of technology among underserved populations to reduce the risk of furthering inequities in care for these individuals. Research that is co-produced with underserved populations according to their specific needs on integrating TBI and MHSU care is urgently needed. Finally, sex and gender considerations must be incorporated in all research, including the reporting of data stratified by sex or gender. Apart from case studies, which predominantly comprised participants of a single sex or gender, none of the articles in this review reported sex- or gender-stratified data. Furthermore, two studies reported findings on men and women but were described as ‘sex’ [55, 56] while 12 studies reported findings on males and females but were described as ‘gender’ [69, 74–76, 85, 86, 92, 95, 98, 103–105]. Clear reporting of sex and gender data are needed to inform gender transformative integrated care.

### Strengths and limitations

This review is the first, to the best of our knowledge, to systematically review the peer-reviewed literature to identify existing integrated care for TBI and MHSU. The primary search strategy was supplemented with a secondary search to capture articles that describe integrated activities but do not explicitly describe their study as such (e.g., screening). Similarly, the title and abstract screen was purposely broad to include articles on care for TBI or MHSU, recognizing that many abstracts may only describe data or refer to TBI or MHSU. Including these articles in the full-text screen reduced the risk of missing relevant articles.

Limitations of this review include the risk of several forms of bias. As non-English language studies were excluded, integrated care in jurisdiction where English is not a primary language may be missed, limiting the comprehensiveness of this review. This review did not include a search of grey literature, meaning that non-peer-reviewed reports from institutions or health systems on their programs or services are missed in this review, which may explain the lack of integration activity at the macro-level. Indeed, notably absent were any studies focusing on strengthening governance and accountability (Strategy 2) or creating an enabling environment (Strategy 5). It is possible that research addressing these two strategies would be captured in organizational or other related grey literature that were not included in the search for this review. We recognize publication bias in this review, as unpublished results and non-peer-reviewed results were excluded, and the risk of biases including but not limited to selection, reporting, information, and performance bias, in studies that integrated treatments or programs for individuals with TBI and MHSU. These biases may contribute to the lack of studies reporting negative outcomes of integrated care. However, given that the goal of this review was to comprehensively summarize all integrated TBI and MHSU care and not to evaluate the impact of the integrated care, we believe the inclusion of study designs such as case studies/series and observational studies, in addition to RCTs, was appropriate. Finally, we acknowledge that the majority of studies included in this review were conducted in the United States and as such, findings may not be directly generalizable to other health systems or settings.

## Conclusion

Taken together, this review provides an overview of the current knowledge base of integrated care for TBI and MHSU, while highlighting areas needing future research. It also provides an evidence-based foundation to inform public health sector mobilization and adaptation of existing integrated care resources for individuals with TBI and MHSU. Integrated TBI and MHSU care already exists across a range of integration activities (e.g., programs, treatments, screening), levels (micro and meso), and types (clinical, service, functional). Opportunities to facilitate the integration of TBI and MHSU care include embedding accommodations to address cognitive challenges experienced by individuals with TBI, including among facilities that are already providing therapy to individuals with MHSU or TBI, to facilitate access to care for individuals with TBI. Multidisciplinary teams, particularly within a single facility, should be explored as it may improve patient outcomes by addressing other sequelae of TBI and MHSU, supporting caregivers by addressing transportation barriers, and promoting education among health professionals so they can be familiar with TBI and MHSU. Finally, research co-produced with individuals with lived experience to understand the benefits and consequences of screening as well as research specifically with underserved populations to enable equitable access to healthcare and research are urgently needed.

## Supporting information

S1 File Search strategies. (PDF)

S1 Table Data extraction table. (PDF)
