# Supplementary material for: A systematic review on integrated care for traumatic brain injury, mental health, and substance use
Source: PLoS One. 2022 Mar 3;17(3):e0264116. doi: 10.1371/journal.pone.0264116 (PMC8893633; doi:10.1371/journal.pone.0264116)
Supplement: S1 File — (PDF) [file pone.0264116.s001.pdf]

SEARCH STRATEGIES

Database: **Ovid MEDLINE: Epub Ahead of Print, In-Process & Other Non-Indexed Citations, Ovid MEDLINE® Daily and Ovid MEDLINE**

Search Strategy:

----

1 exp Brain Injuries/ (62332)

2 exp Brain Injuries, Traumatic/ (9743)

3 exp Brain Concussion/ (7389)

4 Craniocerebral Trauma/ (21314)

5 tbi*2.tw,kf. (22847)

6 mtbi*2.tw,kf. (2223)

7 wrTBI*2.tw,kf. (12)

8 concuss*.tw,kf. (7606)

9 postconcuss*.tw,kf. (1154)

10 ((brain or head* or cerebr* or crani* or skull* or intracran*) adj2 (injur* or trauma* or damag* or wound* or fracture* or contusion*)).tw,kf. (128306)

11 ((brain* or cerebr* or intracerebr* or crani* or intracran* or head* or subdural* or epidural* or extradural*) adj (haematoma* or hematoma* or hemorrhag* or haemorrhag*)).tw,kf. (44556)

12 "shaken baby".tw,kf. (605)

13 or/1-12 (196484)

14 exp Substance-Related Disorders/ (259829)

15 gambling/ (4842)

16 ((drug or substance? or alcohol or opioid? or amphetamine? or cocaine or marijuana or cannabis or phencyclidine or benzodiaz*) adj2 (misuse or abuse* or addict* or depend*)).tw,kf. (108773)

17 gambl???.tw,kf. (8414)

18 (alcoholi* or drinker* or drinking).tw,kf. (177792)

19 or/14-18 (424000)

20 13 and 19 (3854)

21 Mental Health/ (31512)

22 mental disorders/ or exp anxiety disorders/ or exp "bipolar and related disorders"/ or "disruptive, impulse control, and conduct disorders"/ or exp dissociative disorders/ or exp "feeding and eating disorders"/ or exp mood disorders/ or neurodevelopmental disorders/ or anxiety, separation/ or exp "attention deficit and disruptive behavior disorders"/ or child behavior disorders/ or exp child development disorders, pervasive/ or schizophrenia, childhood/ or exp personality disorders/ or exp "schizophrenia spectrum and other psychotic disorders"/ or exp "trauma and stressor related disorders"/ (586165)

23 Depression/ (103355)

24 exp Depressive Disorder, Major/ (26327)

25 exp Depressive Disorder/ (99948)

26 ((mental or psych*) adj (health* or illness* or disease* or condition* or disorder?)).tw,kf. (241708)
27 anxiety.tw,kf. (163525)
28 PTSD.tw,kf. (20669)
29 post-traumatic stress disorder?.tw,kf. (9534)
30 obsessive compulsive.tw,kf. (15892)
31 (depression? or depressed or depressiv* or MDD).tw,kf. (392484)
32 Schizophreni*.tw,kf. (116454)
33 bipolar.tw,kf. (56704)
34 conduct disorder?.tw,kf. (4279)
35 attention deficit.tw,kf. (24841)
36 hoard*.tw,kf. (1461)
37 or/21-36 (1084130)
38 13 and 37 (13021)
39 20 or 38 (15916)
40 exp "Delivery of Health Care, Integrated"/ (11218)
41 ((vertical or horizontal or integrat* or coordinat* or co-ordinat* or link*) and (care or health care or healthcare or service? or treatment? or therap* or psychotherap* or program*)).tw,kf. (452323)
42 (integrat* adj3 (care or health care or healthcare or service? or treatment? or therap* or psychotherap* or program*)).tw,kf. (33149)
43 (connect* adj3 (care or health care or healthcare or service? or treatment? or therap* or psychotherap* or program*)).tw,kf. (4447)
44 (coordinat* adj3 (care or health care or healthcare or service? or treatment? or therap* or psychotherap* or program*)).tw,kf. (14002)
45 or/40-44 (461475)
46 39 and 45 (574)
47 (barrier? or hurdle? or obstacle? or challeng* or incentiv* or obstruct* or refusal? or impedim* or promot* or facilitat* or support* or enabl* or cause? or reason? or encourag* or predict* or hinder* or willingness).tw,kf. (6647308)
48 ((seek? or seeking) adj3 (help* or treatment? or care)).tw,kf. (31739)
49 47 or 48 (6661497)
50 Community health services/ (30003)
51 exp Primary Health Care/ (139690)
52 Patient participation/ (22821)
53 Patient Care Team/ (60979)
54 case management/ (9554)
55 critical pathways/ (6019)
56 exp "continuity of patient care"/ (218438)
57 health education/ (57952)
58 health promotion/ (67033)
59 health planning/ (21288)

60 patient care management/ (3219)
61 comprehensive health care/ (6407)
62 exp "delivery of health care"/ (978727)
63 disease management/ (30690)
64 critical pathways/ (6019)
65 patient-centered care/ (16429)
66 exp "outcome and process assessment (health care)"/ (977104)
67 exp program evaluation/ (68936)
68 quality assurance, health care/ (54119)
69 ((interdisciplin* or inter-disciplin* or interprofession* or inter-profession* or multidisciplin* or multi-disciplin* or multiprofession* or multi-profession*) adj3 (team or care or health care or healthcare or service? or treatment? or therap* or program*)).tw,kf. (38696)
70 (engagement or engage or engaging or empower* or participat*).tw,kf. (569356)
71 (governan* or accountab*).tw,kf. (25773)
72 (model adj2 care).tw,kf. (8943)
73 (educat* or screen* or train* or transition* or evaluat* or coordinat*).tw,kf. (4766860)
74 or/50-73 (6666001)
75 39 and 49 and 74 (2505)
76 46 or 75 (2867)
77 limit 76 to english language (2676)
78 77 not (exp animals/ not exp humans/) (2521)

****

Database: **Cochrane Central Register of Controlled Trials** <2014 to Present>
Search Strategy:
----
1 exp Brain Injuries/ (1732)
2 exp Brain Injuries, Traumatic/ (1732)
3 exp Brain Concussion/ (241)
4 Craniocerebral Trauma/ (283)
5 tbi*2.tw,kf. (2040)
6 mtbi*2.tw,kf. (250)
7 wrTBI*2.tw,kf. (0)
8 concuss*.tw,kf. (408)
9 postconcuss*.tw,kf. (102)
10 ((brain or head* or cerebr* or crani* or skull* or intracran*) adj2 (injur* or trauma* or damag* or wound* or fracture* or contusion*)).tw,kf. (6161)
11 ((brain* or cerebr* or intracerebr* or crani* or intracran* or head* or subdural* or epidural* or extradural*) adj (haematoma* or hematoma* or hemorrhag* or haemorrhag*)).tw,kf. (3526)

12 "shaken baby".tw,kf. (9)
13 or/1-12 (10514)
14 exp Substance-Related Disorders/ (13128)
15 gambling/ (298)
16 ((drug or substance? or alcohol or opioid? or amphetamine? or cocaine or marijuana or cannabis or phencyclidine or benzodiaz*) adj2 (misuse or abuse* or addict* or depend*)).tw,kf. (10967)
17 gambl???.tw,kf. (623)
18 (alcoholi* or drinker* or drinking).tw,kf. (11000)
19 or/14-18 (26428)
20 13 and 19 (185)
21 Mental Health/ (1123)
22 mental disorders/ or exp anxiety disorders/ or exp "bipolar and related disorders"/ or "disruptive, impulse control, and conduct disorders"/ or exp dissociative disorders/ or exp "feeding and eating disorders"/ or exp mood disorders/ or neurodevelopmental disorders/ or anxiety, separation/ or exp "attention deficit and disruptive behavior disorders"/ or child behavior disorders/ or exp child development disorders, pervasive/ or schizophrenia, childhood/ or exp personality disorders/ or exp "schizophrenia spectrum and other psychotic disorders"/ or exp "trauma and stressor related disorders"/ (26375)
23 Depression/ (9417)
24 exp Depressive Disorder, Major/ (3972)
25 exp Depressive Disorder/ (10136)
26 ((mental or psych*) adj (health* or illness* or disease* or condition* or disorder?)).tw,kf. (17190)
27 anxiety.tw,kf. (28440)
28 PTSD.tw,kf. (2944)
29 post-traumatic stress disorder?.tw,kf. (994)
30 obsessive compulsive.tw,kf. (1950)
31 (depression? or depressed or depressiv* or MDD).tw,kf. (53605)
32 Schizophreni*.tw,kf. (12510)
33 bipolar.tw,kf. (5592)
34 conduct disorder?.tw,kf. (313)
35 attention deficit.tw,kf. (2995)
36 hoard*.tw,kf. (53)
37 or/21-36 (103330)
38 13 and 37 (847)
39 20 or 38 (939)
40 exp "Delivery of Health Care, Integrated"/ (314)
41 ((vertical or horizontal or integrat* or coordinat* or co-ordinat* or link*) and (care or health care or healthcare or service? or treatment? or therap* or psychotherap* or program*)).tw,kf. (29535)
42 (integrat* adj3 (care or health care or healthcare or service? or treatment? or therap* or psychotherap* or program*)).tw,kf. (4849)

43 (connect* adj3 (care or health care or healthcare or service? or treatment? or therap* or psychotherap* or program*)).tw,kf. (688)
44 (coordinat* adj3 (care or health care or healthcare or service? or treatment? or therap* or psychotherap* or program*)).tw,kf. (1369)
45 or/40-44 (30171)
46 39 and 45 (88)
47 (barrier? or hurdle? or obstacle? or challeng* or incentiv* or obstruct* or refusal? or impedim* or promot* or facilitat* or support* or enabl* or cause? or reason? or encourag* or predict* or hinder* or willingness).tw,kf. (311112)
48 ((seek? or seeking) adj3 (help* or treatment? or care)).tw,kf. (3523)
49 47 or 48 (312722)
50 Community health services/ (898)
51 exp Primary Health Care/ (4481)
52 Patient participation/ (1122)
53 Patient Care Team/ (1538)
54 case management/ (655)
55 critical pathways/ (171)
56 exp "continuity of patient care"/ (579)
57 health education/ (3492)
58 health promotion/ (4893)
59 health planning/ (34)
60 patient care management/ (119)
61 comprehensive health care/ (67)
62 exp "delivery of health care"/ (38828)
63 disease management/ (804)
64 critical pathways/ (171)
65 patient-centered care/ (450)
66 exp "outcome and process assessment (health care)"/ (127732)
67 exp program evaluation/ (5433)
68 quality assurance, health care/ (612)
69 ((interdisciplin* or inter-disciplin* or interprofession* or inter-profession* or multidisciplin* or multi-disciplin* or multiprofession* or multi-profession*) adj3 (team or care or health care or healthcare or service? or treatment? or therap* or program*)).tw,kf. (3163)
70 (engagement or engage or engaging or empower* or participat*).tw,kf. (73190)
71 (governan* or accountab*).tw,kf. (574)
72 (model adj2 care).tw,kf. (1606)
73 (educat* or screen* or train* or transition* or evaluat* or coordinat*).tw,kf. (428554)
74 or/50-73 (543646)
75 39 and 49 and 74 (292)
76 46 or 75 (336)

77 limit 76 to english language (187)

****

Database: **Embase Classic+Embase**
Search Strategy:
----
1 exp brain injury/ (170129)
2 head injury/ (50510)
3 tbi*2.tw. (37286)
4 mtbi*2.tw. (3458)
5 wrTBI*2.tw. (16)
6 concuss*.tw. (10071)
7 postconcuss*.tw. (1412)
8 ((brain or head* or cerebr* or crani* or skull* or intracran*) adj2 (injur* or trauma* or damag* or wound* or fracture* or contusion*)).tw. (173261)
9 ((brain* or cerebr* or intracerebr* or crani* or intracran* or head* or subdural* or epidural* or extradural*) adj (haematoma* or hematoma* or hemorrhag* or haemorrhag*)).tw. (64309)
10 "shaken baby".tw. (743)
11 or/1-10 (329144)
12 addiction/ (51840)
13 exp drug dependence/ (227621)
14 pathological gambling/ (5577)
15 ((drug or substance? or alcohol or opioid? or amphetamine? or cocaine or marijuana or cannabis or phencyclidine or benzodiaz*) adj2 (misuse or abuse* or addict* or depend*)).tw. (146297)
16 gambl???.tw. (10917)
17 (alcoholi* or drinker* or drinking).tw. (248964)
18 or/12-17 (514147)
19 11 and 18 (6730)
20 exp mental health/ (132537)
21 mental disease/ (222219)
22 exp anxiety disorder/ (211380)
23 exp bipolar disorder/ (57278)
24 behavior disorder/ or attention deficit disorder/ or disruptive behavior/ or drug seeking behavior/ or impulse control disorder/ (107231)
25 exp dissociative disorder/ (8226)
26 exp eating disorder/ (47110)
27 exp mood disorder/ (461687)
28 exp autism/ (57648)
29 exp schizophrenia/ (181763)

30 exp personality disorder/ (59926)
31 exp psychosis/ (284664)
32 ((mental or psych*) adj (health* or illness* or disease* or condition* or disorder?)).tw. (304770)
33 PTSD.tw. (25839)
34 post-traumatic stress disorder?.tw. (12129)
35 obsessive compulsive.tw. (20726)
36 (depression? or depressed or depressiv* or MDD).tw. (545145)
37 Schizophreni*.tw. (158068)
38 bipolar.tw. (81682)
39 conduct disorder?.tw. (5392)
40 attention deficit.tw. (31286)
41 hoard*.tw. (1814)
42 or/20-41 (1531303)
43 11 and 42 (29736)
44 19 or 43 (34158)
45 integrated health care system/ (9873)
46 ((vertical or horizontal or integrat* or coordinat* or co-ordinat* or link*) and (care or health care or healthcare or service? or treatment? or therap* or psychotherap* or program*)).tw. (624215)
47 (integrat* adj3 (care or health care or healthcare or service? or treatment? or therap* or psychotherap* or program*)).tw. (44675)
48 (connect* adj3 (care or health care or healthcare or service? or treatment? or therap* or psychotherap* or program*)).tw. (6653)
49 (coordinat* adj3 (care or health care or healthcare or service? or treatment? or therap* or psychotherap* or program*)).tw. (19801)
50 or/45-49 (634621)
51 44 and 50 (1382)
52 (barrier? or hurdle? or obstacle? or challeng* or incentiv* or obstruct* or refusal? or impedim* or promot* or facilitat* or support* or enabl* or cause? or reason? or encourag* or predict* or hinder* or willingness).tw. (8662026)
53 ((seek? or seeking) adj3 (help* or treatment? or care)).tw. (40702)
54 52 or 53 (8679961)
55 exp community care/ (116494)
56 exp primary health care/ (147878)
57 patient participation/ (23829)
58 exp patient care/ (719197)
59 clinical pathway/ (7778)
60 exp health education/ (297823)
61 health care planning/ (94317)
62 exp health care delivery/ (2870808)
63 disease management/ (19050)

64 outcome assessment/ (432109)
65 exp health care quality/ (2772612)
66 exp program evaluation/ (17544)
67 ((interdisciplin* or inter-disciplin* or interprofession* or inter-profession* or multidisciplin* or multi-disciplin* or multiprofession* or multi-profession*) adj3 (team or care or health care or healthcare or service? or treatment? or therap* or program*)).tw. (64305)
68 (engagement or engage or engaging or empower* or participat*).tw. (743424)
69 (governan* or accountab*).tw. (31041)
70 (model adj2 care).tw. (12583)
71 (educat* or screen* or train* or transition* or evaluat* or coordinat*).tw. (6328902)
72 or/55-71 (10373343)
73 44 and 54 and 72 (7037)
74 51 or 73 (7854)
75 limit 74 to english language (7426)
76 75 not ((exp animals/ or exp animal experimentation/ or nonhuman/) not exp human/) (6887)
77 limit 76 to conference abstracts (2365)
78 76 not 77 (4522)
79 78 not medline.cr. (4055)

****

Database: **PsycINFO**
Search Strategy:
----
1 exp traumatic brain injury/ (17391)
2 exp head injuries/ (5923)
3 tbi*2.tw. (9703)
4 mtbi*2.tw. (1562)
5 wrTBI*2.tw. (8)
6 concuss*.tw. (2850)
7 postconcuss*.tw. (796)
8 ((brain or head* or cerebr* or crani* or skull* or intracran*) adj2 (injur* or trauma* or damag* or wound* or fracture* or contusion*)).tw. (45163)
9 ((brain* or cerebr* or intracerebr* or crani* or intracran* or head* or subdural* or epidural* or extradural*) adj (haematoma* or hematoma* or hemorrhag* or haemorrhag*)).tw. (3211)
10 "shaken baby".tw. (185)
11 or/1-10 (49487)
12 exp drug abuse/ (105296)
13 "substance use disorder"/ (5723)
14 addiction/ (9744)

15 exp gambling/ (7117)

16 ((drug or substance? or alcohol or opioid? or amphetamine? or cocaine or marijuana or cannabis or phencyclidine or benzodiaz*) adj2 (misuse or abuse* or addict* or depend*)).tw. (95808)

17 gambl???.tw. (11158)

18 (alcoholi* or drinker* or drinking).tw. (71401)

19 or/12-18 (189802)

20 11 and 19 (1834)

21 mental health/ (57737)

22 mental disorders/ (78899)

23 exp anxiety disorders/ (77257)

24 exp affective disorders/ (151634)

25 exp impulse control disorders/ (1034)

26 conduct disorder/ (4163)

27 behavior disorders/ (9073)

28 exp dissociative disorders/ (5069)

29 exp eating disorders/ (28488)

30 neurodevelopmental disorders/ (1969)

31 autism spectrum disorders/ (38498)

32 exp separation anxiety/ (1529)

33 exp attention deficit disorder/ (24934)

34 exp psychosis/ (109127)

35 exp personality disorders/ (33271)

36 exp posttraumatic stress disorder/ (29576)

37 ((mental or psych*) adj (health* or illness* or disease* or condition* or disorder?)).tw. (306679)

38 anxiety.tw. (180502)

39 PTSD.tw. (29697)

40 post-traumatic stress disorder?.tw. (9443)

41 obsessive compulsive.tw. (19372)

42 (depression? or depressed or depressiv* or MDD).tw. (278508)

43 Schizophreni*.tw. (118127)

44 bipolar.tw. (37311)

45 conduct disorder?.tw. (7217)

46 attention deficit.tw. (29043)

47 hoard*.tw. (1855)

48 or/21-47 (887448)

49 11 and 48 (9062)

50 20 or 49 (10045)

51 integrated services/ (3216)

52 ((vertical or horizontal or integrat* or coordinat* or co-ordinat* or link*) and (care or health care or healthcare or service? or treatment? or therap* or psychotherap* or program*)).tw. (152611)

53 (integrat* adj3 (care or health care or healthcare or service? or treatment? or therap* or psychotherap* or program*)).tw. (20889)
54 (connect* adj3 (care or health care or healthcare or service? or treatment? or therap* or psychotherap* or program*)).tw. (2818)
55 (coordinat* adj3 (care or health care or healthcare or service? or treatment? or therap* or psychotherap* or program*)).tw. (5590)
56 or/51-55 (155650)
57 50 and 56 (576)
58 Treatment Barriers/ (4168)
59 (barrier? or hurdle? or obstacle? or challeng* or incentiv* or obstruct* or refusal? or impedim* or promot* or facilitat* or support* or enabl* or cause? or reason? or encourag* or predict* or hinder* or willingness).tw. (1644346)
60 ((seek? or seeking) adj3 (help* or treatment? or care)).tw. (24738)
61 or/58-60 (1655245)
62 exp community services/ (31008)
63 primary health care/ (16770)
64 health care services/ (40379)
65 "continuum of care"/ (1628)
66 health care delivery/ (19878)
67 interdisciplinary treatment approach/ (6871)
68 client participation/ (1850)
69 exp case management/ (3343)
70 exp managed care/ (4298)
71 client centered therapy/ (3105)
72 exp "quality of services"/ (17955)
73 exp health education/ or client education/ (20686)
74 health promotion/ (22605)
75 exp treatment planning/ (6202)
76 disease management/ (6121)
77 exp program evaluation/ (19435)
78 ((interdisciplin* or inter-disciplin* or interprofession* or inter-profession* or multidisciplin* or multi-disciplin* or multiprofession* or multi-profession*) adj3 (team or care or health care or healthcare or service? or treatment? or therap* or program*)).tw. (11242)
79 (engagement or engage or engaging or empower* or participat*).tw. (374143)
80 (governan* or accountab*).tw. (25304)
81 (model adj2 care).tw. (3435)
82 (educat* or screen* or train* or transition* or evaluat* or coordinat*).tw. (1245279)
83 or/62-82 (1562608)
84 50 and 61 and 83 (1650)
85 57 or 84 (2011)

86 limit 85 to english language (1934)

87 limit 86 to animal (115)

88 limit 87 to human (33)

89 86 not (87 not 88) (1852)

90 limit 89 to ("0200 book" or "0240 authored book" or "0280 edited book" or "0300 encyclopedia") (458)

91 89 not 90 (1394)

****

Database: **CINAHL**

#	Query	Limiters/Expanders	Results
S81	S80	Limiters - English Language<br>Search modes - Boolean/Phrase	1,242
S80	S52 OR S79	Search modes - Boolean/Phrase	1,250
S79	S45 AND S55 AND S78	Search modes - Boolean/Phrase	1,123
S78	S56 OR S57 OR S58 OR S59 OR S60 OR S61 OR S62 OR S63 OR S64 OR S65 OR S66 OR S67 OR S68 OR S69 OR S70 OR S71 OR S72 OR S73 OR S74 OR S75 OR S76 OR S77	Search modes - Boolean/Phrase	1,628,534
S77	(educat* or screen* or train* or transition* or evaluat* or coordinat*)	Search modes - Boolean/Phrase	1,128,349
S76	(model n2 care)	Search modes - Boolean/Phrase	11,356
S75	(governan* or accountab*)	Search modes - Boolean/Phrase	24,583
S74	(engagement or engage or engaging or empower* or participat*)	Search modes - Boolean/Phrase	175,276
S73	((interdisciplin* or inter-disciplin* or interprofession* or inter-profession* or multidisciplin* or multi-disciplin* or multiprofession* or multi-profession*) n3 (team or care or health care or healthcare or service* or treatment* or therap* or program*))	Search modes - Boolean/Phrase	39,697
S72	(MH "Quality of Health Care+")	Search modes - Boolean/Phrase	413,487
S71	(MH "Program Development+")	Search modes - Boolean/Phrase	51,590
S70	(MH "Outcome Assessment")	Search modes - Boolean/Phrase	23,937
S69	(MH "Process Assessment (Health Care)+")	Search modes - Boolean/Phrase	5,750
S68	(MH "Patient Centered Care")	Search modes - Boolean/Phrase	18,429
S67	(MH "Disease Management+")	Search modes - Boolean/Phrase	11,760
S66	(MH "Health Care Delivery+")	Search modes - Boolean/Phrase	210,880
S65	(MH "Patient Care Plans+")	Search modes - Boolean/Phrase	6,852
S64	(MH "Health Promotion")	Search modes - Boolean/Phrase	39,646
S63	(MH "Health Education+")	Search modes - Boolean/Phrase	88,614
S62	(MH "Continuity of Patient Care+")	Search modes - Boolean/Phrase	12,938
S61	(MH "Critical Path")	Search modes - Boolean/Phrase	3,710
S60	(MH "Case Management")	Search modes - Boolean/Phrase	13,403
S59	(MH "Multidisciplinary Care Team")	Search modes - Boolean/Phrase	28,529
S58	(MH "Consumer Participation")	Search modes - Boolean/Phrase	12,214
S57	(MH "Primary Health Care")	Search modes - Boolean/Phrase	38,581
S56	(MH "Community Health Services")	Search modes - Boolean/Phrase	13,940
S55	S53 OR S54	Search modes - Boolean/Phrase	903,092
S54	((seek or seeks or seeking) n3 (help* or treatment* or care))	Search modes - Boolean/Phrase	13,303

S53	(barrier* or hurdle* or obstacle* or challeng* or incentiv* or obstruct* or refusal* or impedim* or promot* or facilitat* or support* or enabl* or cause* or reason* or encourag* or predict* or hinder* or willingness)	Search modes - Boolean/Phrase	897,161
S52	S45 AND S51	Search modes - Boolean/Phrase	240
S51	S46 OR S47 OR S48 OR S49 OR S50	Search modes - Boolean/Phrase	112,403
S50	(coordinat* n3 (care or health care or healthcare or service* or treatment* or therap* or psychotherap* or program*))	Search modes - Boolean/Phrase	6,957
S49	(connect* n3 (care or health care or healthcare or service* or treatment* or therap* or psychotherap* or program*))	Search modes - Boolean/Phrase	2,263
S48	(integrat* n3 (care or health care or healthcare or service* or treatment* or therap* or psychotherap* or program*))	Search modes - Boolean/Phrase	19,214
S47	((vertical or horizontal or integrat* or coordinat* or co-ordinat* or link*) and (care or health care or healthcare or service* or treatment* or therap* or psychotherap* or program*))	Search modes - Boolean/Phrase	110,503
S46	(MH "Health Care Delivery, Integrated")	Search modes - Boolean/Phrase	6,478
S45	S19 OR S44	Search modes - Boolean/Phrase	4,356
S44	S12 AND S43	Search modes - Boolean/Phrase	3,562
S43	S20 OR S21 OR S22 OR S23 OR S24 OR S25 OR S26 OR S27 OR S28 OR S29 OR S30 OR S31 OR S32 OR S33 OR S34 OR S35 OR S36 OR S37 OR S38 OR S39 OR S40 OR S41 OR S42	Search modes - Boolean/Phrase	295,552
S42	hoard*	Search modes - Boolean/Phrase	279
S41	attention deficit	Search modes - Boolean/Phrase	8,798
S40	conduct disorder*	Search modes - Boolean/Phrase	762
S39	bipolar	Search modes - Boolean/Phrase	7,852
S38	Schizophreni*	Search modes - Boolean/Phrase	13,979
S37	(depression* or depressed or depressiv* or MDD)	Search modes - Boolean/Phrase	92,716
S36	obsessive compulsive	Search modes - Boolean/Phrase	2,839
S35	post-traumatic stress disorder*	Search modes - Boolean/Phrase	2,651
S34	PTSD	Search modes - Boolean/Phrase	4,813
S33	anxiety	Search modes - Boolean/Phrase	48,239
S32	((mental or psych*) n1 (health* or illness* or disease* or condition* or disorder*))	Search modes - Boolean/Phrase	150,733
S31	(MH "Stress Disorders, Post-Traumatic")	Search modes - Boolean/Phrase	11,557
S30	(MH "Attention Deficit Hyperactivity Disorder") OR (MH "Mental Disorders Diagnosed in Childhood") OR (MH "Child Behavior Disorders+") OR (MH "Child Development Disorders+") OR (MH "Child Development Disorders, Pervasive+") OR (MH "Feeding and Eating Disorders of Childhood+")	Search modes - Boolean/Phrase	28,519
S29	(MH "Separation Anxiety")	Search modes - Boolean/Phrase	382
S28	(MH "Depression+")	Search modes - Boolean/Phrase	59,957
S27	(MH "Affective Disorders+")	Search modes - Boolean/Phrase	63,151
S26	(MH "Eating Disorders+")	Search modes - Boolean/Phrase	11,050
S25	(MH "Dissociative Disorders+")	Search modes - Boolean/Phrase	665
S24	(MH "Personality Disorders+")	Search modes - Boolean/Phrase	6,356

S23	(MH "Affective Disorders, Psychotic+") OR (MH "Psychotic Disorders") OR (MH "Schizoaffective Disorder") OR (MH "Schizophrenia+")	Search modes - Boolean/Phrase	19,775
S22	(MH "Anxiety Disorders+")	Search modes - Boolean/Phrase	21,620
S21	(MH "Mental Disorders")	Search modes - Boolean/Phrase	33,506
S20	(MH "Mental Health")	Search modes - Boolean/Phrase	18,412
S19	S12 AND S18	Search modes - Boolean/Phrase	1,098
S18	S13 OR S14 OR S15 OR S16 OR S17	Search modes - Boolean/Phrase	122,473
S17	alcoholi* or drinker* or drinking	Search modes - Boolean/Phrase	37,825
S16	(gamble* or gambling*)	Search modes - Boolean/Phrase	1,914
S15	((drug or substance* or alcohol or opioid* or amphetamine* or cocaine or marijuana or cannabis or phencyclidine or benzodiaz*) n2 (misuse or abuse* or addict* or depend*))	Search modes - Boolean/Phrase	56,272
S14	(MH "Gambling")	Search modes - Boolean/Phrase	1,265
S13	(MH "Substance Use Disorders+")	Search modes - Boolean/Phrase	93,525
S12	S1 OR S2 OR S3 OR S4 OR S5 OR S6 OR S7 OR S8 OR S9 OR S10 OR S11	Search modes - Boolean/Phrase	41,008
S11	shaken baby	Search modes - Boolean/Phrase	392
S10	((brain* or cerebr* or intracerebr* or crani* or intracran* or head* or subdural* or epidural* or extradural*) n1 (haematoma* or hematoma* or hemorrhag* or haemorrhag*))	Search modes - Boolean/Phrase	9,061
S9	((brain or head* or cerebr* or crani* or skull* or intracran*) n2 (injur* or trauma* or damag* or wound* or fracture* or contusion*))	Search modes - Boolean/Phrase	30,635
S8	postconcuss*	Search modes - Boolean/Phrase	741
S7	concuss*	Search modes - Boolean/Phrase	3,360
S6	wrTBI*	Search modes - Boolean/Phrase	7
S5	mTBI*	Search modes - Boolean/Phrase	614
S4	TBI*	Search modes - Boolean/Phrase	5,222
S3	(MH "Head Injuries")	Search modes - Boolean/Phrase	4,698
S2	(MH "Brain Concussion+")	Search modes - Boolean/Phrase	2,888
S1	(MH "Brain Injuries+")	Search modes - Boolean/Phrase	18,914

Database: **Sociological Abstracts**

(noft(tbi* OR mtbi* OR concuss* OR postconcuss* OR "shaken baby") OR noft(((brain OR head* OR cerebr* OR crani* OR skull* OR intracran*) NEAR/2 (injur* OR trauma* OR damag* OR wound* OR fracture* OR contusion*)))) AND (noft(((drug OR substance? OR alcohol OR opioid? OR amphetamine? OR cocaine OR marijuana OR cannabis OR phencyclidine OR benzodiaz*) NEAR/2 (misuse OR abuse* OR addict* OR depend*))) OR noft(gambl???) OR noft((alcoholi* OR drinker* OR drinking)) OR noft(((mental OR psych*) NEAR/1 (health* OR illness* OR disease* OR condition* OR disorder?))) OR noft(anxiety OR PTSD OR "post-traumatic stress" OR "obsessive compulsive" OR depression? OR depressed OR depressiv* OR MDD OR Schizophreni* OR "attention deficit" OR hoard*) OR noft("conduct disorder" OR "conduct disorders"))

Limits applied

- Language: English
- Exclude: Books; Magazines

Database: **ProQuest Dissertations & Theses Global**

(((noft(tbi* OR mtbi* OR concuss* OR postconcuss* OR "shaken baby") OR noft(((brain OR head* OR cerebr* OR crani* OR skull* OR intracran*) NEAR/2 (injur* OR trauma* OR damag* OR wound* OR fracture* OR contusion*)))) AND (noft(((drug OR substance? OR alcohol OR opioid? OR amphetamine? OR cocaine OR marijuana OR cannabis OR phencyclidine OR benzodiaz*) NEAR/2 (misuse OR abuse* OR addict* OR depend*))) OR noft(gambl???) OR noft((alcoholi* OR drinker* OR drinking)) OR noft(((mental OR psych*) NEAR/1 (health* OR illness* OR disease* OR condition* OR disorder?))) OR noft(anxiety OR PTSD OR "post-traumatic stress" OR "obsessive compulsive" OR depression? OR depressed OR depressiv* OR MDD OR Schizophreni* OR "attention deficit" OR hoard*) OR noft("conduct disorder" OR "conduct disorders"))) AND ((noft(barrier* OR hurdle* OR obstacle* OR challeng* OR incentiv* OR obstruct* OR refusal* OR impedim* OR promot* OR facilitat* OR support* OR enabl* OR cause* OR reason* OR encourag* OR predict* OR hinder* OR willingness) OR noft(seek* NEAR/3 help*) OR noft(seek* NEAR/3 treatment*) OR noft(seek* NEAR/3 care)) AND (noft((interdisciplin* OR inter-disciplin* OR interprofession* OR inter-profession* OR multidisciplin* OR multi-disciplin* OR multiprofession* OR multi-profession*)) OR noft(engagement OR engage OR engaging OR empower* OR participat*) OR noft(governan* OR accountab*) OR noft(model NEAR/2 care) OR noft(educat* OR screen* OR train* OR transition* OR evaluat* OR coordinat*)))) OR (((noft(tbi* OR mtbi* OR concuss* OR postconcuss* OR "shaken baby") OR noft(((brain OR head* OR cerebr* OR crani* OR skull* OR intracran*) NEAR/2 (injur* OR trauma* OR damag* OR wound* OR fracture* OR contusion*)))) AND (noft(((drug OR substance? OR alcohol OR opioid? OR amphetamine? OR cocaine OR marijuana OR cannabis OR phencyclidine OR benzodiaz*) NEAR/2 (misuse OR abuse* OR addict* OR depend*))) OR noft(gambl???) OR noft((alcoholi* OR drinker* OR drinking)) OR noft(((mental OR psych*) NEAR/1 (health* OR illness* OR disease* OR condition* OR disorder?))) OR noft(anxiety OR PTSD OR "post-traumatic stress" OR "obsessive compulsive" OR depression? OR depressed OR depressiv* OR MDD OR Schizophreni* OR "attention deficit" OR hoard*) OR noft("conduct disorder" OR "conduct disorders"))) AND ((noft((vertical OR horizontal OR integrat* OR coordinat* OR co-ordinat* OR link*)) AND noft((care OR health care OR healthcare OR service* OR treatment* OR therap* OR psychotherap* OR program*))) OR (noft((integrat*) NEAR/3 (care)) OR noft((integrat*) NEAR/3 ("health care")) OR noft((integrat*) NEAR/3 (healthcare)) OR noft((integrat*) NEAR/3 (healthcare)) OR noft((integrat*) NEAR/3 (service*)) OR noft((integrat*) NEAR/3 (treatment*)) OR noft((integrat*) NEAR/3 (therap*)) OR noft((integrat*) NEAR/3 (psychotherap*)) OR noft((integrat*) NEAR/3 (program*)) OR noft((coordinat*) NEAR/3 (care)) OR noft((coordinat*) NEAR/3 ("health care")) OR noft((coordinat*) NEAR/3 (healthcare)) OR noft((coordinat*) NEAR/3 (healthcare)) OR noft((coordinat*) NEAR/3 (service*)) OR noft((coordinat*) NEAR/3 (treatment*)) OR noft((coordinat*) NEAR/3 (therap*)) OR noft((coordinat*) NEAR/3 (psychotherap*)) OR noft((coordinat*) NEAR/3 (program*)) OR noft((connect*) NEAR/3 (care)) OR noft((connect*) NEAR/3 ("health care")) OR noft((connect*) NEAR/3 (healthcare)) OR noft((connect*) NEAR/3 (healthcare)) OR noft((connect*) NEAR/3 (service*)) OR noft((connect*) NEAR/3 (treatment*)) OR noft((connect*) NEAR/3 (therap*)) OR noft((connect*) NEAR/3 (psychotherap*)) OR noft((connect*) NEAR/3 (program*)))))Limits applied

Limits applied:

- Language: English
