# Supplementary material for: A systematic review on integrated care for traumatic brain injury, mental health, and substance use
Source: PLoS One. 2022 Mar 3;17(3):e0264116. doi: 10.1371/journal.pone.0264116 (PMC8893633; doi:10.1371/journal.pone.0264116)
Supplement: S1 Table — (PDF) [file pone.0264116.s002.pdf]

Supplementary Table 2. Study Characteristics and Risk of Bias Assessment

Study (Author, Year, Country)	Study Design & Objective	Sample Characteristics	TBI and MHSU Information		Integration Information				WHO Strategic Approach	Facilitator	Barrier	Risk of Bias Assessment
			TBI Severity/Time Since Injury (Mean ± SD, Years, Unless Specified)	Type of MHSU/Time Since Dx (Mean ± SD, Years, Unless Specified)	Activity	Description	Level	Type				
Ahmed et al., 2016<br><br>United States	Case Study<br><br>Integrated model of care to address physical, cognitive, and psychiatric needs among individuals w/TBI	N=1<br>- Gender: Man<br>- Age [Year]: 31 at time of tx; 24 at time of TBI<br>- Marital Status: Divorced<br>- Education: GED	Severe/7 years	ADHD/ Elementary school; MDD/NR	Program	Program of Enhanced Psychiatric Services (PEP)<br>(Outpatient psychiatric brain injury clinic)<br>- Individual and gp psychotherapy + pharmacotherapy<br>- Weekly multidisciplinary team meetings<br>- Individualized tx plans<br>- Family meetings	Meso	Service	1.1<br>1.4	- Integrating family members<br>- Staff experienced in managing behavioural sxs from TBI	- Geographic distance b/t PEP and neurorehabilitation outpatient program	- Selection<br>- Reporting<br>- Information
Albrecht et al., 2017<br><br>United States	Qualitative<br><br>Explore perception of barriers and facilitators to dx and tx of MHSU after TBI	N=33 (10 HCP; 18 patients; 5 caregivers)<br>- Gender [Men]: 83% Patients; 40% Caregivers; HCP NR	Mild-Severe/<br>18 mos-5 years	Depression; Anxiety; PTSD/NR	Screening, Treatment	Dx and tx of MHSU after TBI	Meso	Clinical	4	- Positive and engaged caregiver support	- Lack of education about TBI and MHSU among patients, HCP, and caregivers<br>- Lack of experience with TBI and MHSU among HCP --> reluctance to dx, which delays tx<br>- polypharmacy --> complicates tx of TBI and MHSU<br>- Limited access to care due to insurance, transportation, income, and availability of providers	N/A
Andrew et al., 2017<br><br>South Africa	Cohort<br><br>Incorporate questions re: cognition and MHSU sxs to TBI screening tool to identify patients for referral	N=47<br>- Sex [Males]: 94%<br>- Age [Years]: 16 to 75; mean 35<br>- Employment [Unemployed]: 36% Pre-injury; 11% at time of study	Mild/moderate (N=11); Severe (N=32); Unknown (N=4)/NR	Depression; Anxiety; Hallucinations/NR	Screening	Screening for MHSU among individuals with TBI to improve referral and patient outcome	Meso	Clinical	4	- Interpreter and family/caregiver support	- Communication (due to language and education) --> lack of understanding of some questions<br>- Cultural differences --> difficulty defining depression and personality change	N/A

Ansari et al., 2014<br><br>India	RCT<br><br>Evaluate role of sertraline in depression and impact on QoL after TBI	N=80<br>- Sex [Males]: 100%<br>- Age [Years]: 18-24 (35%); 25-34 (27.5%); 35-44 (16.3%); 45-54 (15%); 55-64 (5%); ≥65 (1.2%)	Mild (54%); Moderate (46%)/ ≥2 weeks - <3 mos (30%);<br>3-6 mos (24%);<br>>6 mos (46%)	Depression/NR	Treatment	Pharmacotherapy (50mg Sertraline/day) over 6 mos	Micro	Clinical	1.1	N/A	N/A	- Selection<br>- Performance<br>- Non-differential misclassification<br>- Information
Ashish et al., 2017<br><br>United States	Case Study<br><br>Describe patient in outpatient behavioural health clinic post-TBI	N=1<br>- Sex: Male<br>- Age [Years]: 14<br>- Ethnicity: Hispanic	Mild/7 weeks (2 additional concussions w/in past 2 years)	Anxiety/NR; Depression/NR; Hallucination/3 weeks post-TBI	Treatment	Individual and family psychotherapy (Acceptance and Commitment Therapy) + vestibular-ocular therapy	Micro	Service	1.1<br>4.1	N/A	N/A	- Reporting<br>- Information
Ashman et al., 2014<br><br>United States	RCT<br><br>Efficacy of CBT vs. SPT in treating depression among individuals w/TBI	N=54 (28 CBT; 26 SPT)<br><br>CBT:<br>- Sex [Males]: 35.7%<br>- Age [Mean ± SD, Years]: 47.5 ± 11<br>- Race/Ethnicity: White (71.4%); Black/African American (10.7%); Hispanic/Latino (10.7%); Other (7.2%)<br>- Marital Status: Single (50%); Married/Live Together (25%); Widowed/Divorced/Separated (25%)<br>- Education: ≤High School/GED (17.9%); Trade School/Some College (21.4%); Bachelor’s Degree (21.4%); ≥Master’s Degree (39.3%)<br>- Income: <$10,000 (25.9%); $10,001–$20,000 (22.2%); $20,001–$60,000 (29.7%); >$60,000 (22.2%)<br><br>SPT:<br>- Sex [Males]: 46.1%<br>- Age [Mean ± SD, Years]: 47.0 ± 10.8<br>- Race/Ethnicity: White (46.1%); Black/African American (15.4%); Hispanic/Latino (23.1%); Other (15.4%)<br>- Marital Status: Single (46.1%); Married/Live Together (23.1%); Widowed/Divorced/Separated (30.8%)<br>- Education: ≤High School/GED (23.1%); Trade School/Some College (42.3%); Bachelor’s Degree (11.5%); ≥Master’s Degree (23.1%)<br>- Income: <$10,000 (23.1%); $10,001–$20,000 (15.4%); $20,001–$60,000 (38.4%); >$60,000 (23.1%)	CBT:<br>Mild (37%); Moderate-Severe (63%)/7.8 ± 13.4<br><br>SPT:<br>Mild (43%); Moderate-Severe (57%)/ 13.2 ± 18.3	Depression/NR	Treatment	Psychotherapy (CBT vs. SPT)<br>- CBT adapted to include accommodations for cognitive challenges	Micro	Clinical	1.1	N/A	N/A	- Small sample<br>- Attrition<br>- Selection

Baig et al., 2019<br><br>United States	Case Control<br><br>Describe model of integrated psychiatric care for PTSD and mTBI	N=100 (50 UC, 50 IC)<br><br>UC:<br>- Age [Mean ± SD, Years]: 33.7 ± 8.24<br>- Gender [Men]: 98%<br>- Marial Status [Not Married]: 25%<br>- Education: HS Diploma (44%); Some College (48%); 4-Year College Degree (8%)<br>- Employment [Unemployed]: 62%<br>- Race: Hispanic/Latino (32%); Caucasian (52%); African American (16%)<br><br>IC:<br>- Age [Mean ± SD, Years]: 34.9 ± 7.8 (IC)<br>- Gender [Men]: 98%<br>- Marital Status [Not Married]: 20%<br>- Education: HS Diploma (36%); Some College (48%); 4-Year College Degree (16%)<br>- Employment [Unemployed]: 50%<br>- Race: Hispanic/Latino (36%); Caucasian (48%); African American (16%)	Mild/NR	PTSD/<br>2.6 ± 2 (UC);<br>1.75 ± 2.3 (IC)	Program	UC:<br>- SW conduct behavioural health evaluation outside of facility<br>- Psychotherapy (most commonly CPT) provided by psychologist at MH outpatient service<br>- Pharmacotherapy, if needed, provided by psychiatrist/physician assistant/advanced practice registered nurse specialist in psychiatry/clinical pharmacy specialist at MH outpatient service<br>- Communications via EMR<br><br>IC:<br>- Psychologist and psychiatrist embedded w/in team at facility<br>- Pharmacotherapy provided by neuropsychiatrist<br>- Psychotherapy (most commonly individual CPT) provided by behavioural therapist<br>- Multidisciplinary team meetings<br>- Alerts for providers via EMR	Meso	Service Functional	1.1<br>4.1	- Integration and collaboration of MHSU care in small, co-locating, and focused polytrauma rehabilitation setting --> improved access to and engagement in tx<br>- Collaboration of psychiatrist and behavioural therapist --> outcome success	N/A	- Referral<br>- Selection<br>- Reporting<br>- Performance
Baig et al., 2019<br><br>United States	Case Series<br><br>Evaluate outcomes of Valproate, Risperidone, and Quetiapine on completion of CPT for PTSD among individuals with TBI	N=50 (8 Valproate; 17 Risperidone; 25 Quetiapine)<br><br>Valproate:<br>- Gender [Men]: 100%<br>- Age [Mean ± SD, Years]: 37 ± 7<br>- Ethnicity [Hispanic]: 38%<br>- Race: White (100%)<br>- Marital Status [Not Married]: 38%<br>- Education: GED (13%); HS Diploma (63%); College Degree (25%)<br>- Employment [Unemployed]: 50%<br><br>Risperidone:<br>- Gender [Men]: 94%<br>- Age [Mean ± SD, Years]: 36 ± 10<br>- Ethnicity [Hispanic]: 35%<br>- Race: Black (24%); White (76%)<br>- Marital Status [Not Married]: 82% Risperidone (82%)<br>- Education: GED (18%); HS Diploma (29%); Some College (29%); College Degree (24%)<br>- Employment [Unemployed]: 65%<br><br>Quetiapine:<br>- Gender [Men]: 96%<br>- Age [Mean ± SD, Years]: 37 ± 8<br>- Ethnicity [Hispanic]: 32%<br>- Race: Asian (8%); Black (20%); White (72%)<br>- Marital Status [Not Married]: 72%<br>- Education: GED (8%); HS Diploma (52%); Some College (28%); College Degree (12%)<br>- Employment [Unemployed]: 68%	mTBI/NR	PTSD/<br>2 ± 3 (Valproate);<br>2 ± 2 (Risperidone);<br>3 ± 8 (Quetiapine)	Treatment	Psychotherapy (CPT and prolonged exposure therapy) + pharmacotherapy (Valproate, Risperidone, Quetiapine)	Meso	Service	1.1	N/A	N/A	- Selection<br>- Information<br>- Attrition

Bay et al., 2017<br><br>United States	Case Study<br><br>Case management model for managing chronic stress and depression after TBI	N=1<br>- Gender: Man<br>- Age [Years]: 90<br>- Employment: Retired<br>- Education: "Well-educated"<br>- Martial Status: Widowed (following 73 year relationship/marriage)	NR/NR	Depression/Prior to TBI	Treatment	Case management with caregiver:<br>- Case manager plans for natural support system, family education, crisis intervention for maintaining QOL; recommends sxs management, reducing loneliness and depression, limiting health service use<br>- Case manager with family to make changes at home and activities for within and outside of home	Micro	Clinical	1.1<br>4.1	- Knowledge of evidence-based practice, protocols, and pathways --> case manager communicate effectively w/interdisciplinary professional and others to engage in proactive patient advocacy	N/A	- Selection<br>- Reporting<br>- Information
Bédard et al., 2014<br><br>Canada	RCT<br><br>Examine efficacy of MBCT for depression among individuals w/TBI	N=76 (38 Tx; 38 Control)<br><br>Tx:<br>- Sex [Males]: 50%<br>- Age [Mean ± SD, Years]: 47.1 ± 12.0<br>- Marital Status: Married/Common law (58%); Single (26%); Separated/Divorced/Widowed (13%); Unknown (3%)<br>- Employment: Employed (37%); Unemployed (21%); Homemaker/Volunteer (11%); Retired (11%); Other (21%)<br>- Education: Elementary (3%); Some Secondary (11%); Completed Secondary (32%); Some Post-Secondary (11%); Completed Post-Secondary (45%)<br><br>Control:<br>- Sex [Males]: 60%<br>- Age [Mean ± SD, Years]: 45.8 ± 14.8<br>- Marital Status: Married/Common law (45%); Single (31%); Separated/Divorced/Widowed (24%); Unknown (0%)<br>- Employment: Employed (24%); Unemployed (26%); Homemaker/Volunteer (0%); Retired (16%); Other (34%)<br>- Education: Elementary (5%); Some Secondary (11%); Completed Secondary (18%); Some Post-Secondary (16%); Completed Post-Secondary (50%)	Tx:<br>NR/4.5 ± 4.1<br><br>Control:<br>NR/4.0 ± 3.5	Depression/NR	Treatment	MBCT:<br>- customized to address issues associated with TBI	Micro	Clinical	1.1	N/A	N/A	- Nondifferential misclassification<br>- Selection<br>- Attrition<br>- Performance
Bombardier et al., 2017<br><br>United States	RCT<br><br>Explore relationship between depression severity and cognition, behaviour, and physical activity among individuals w/TBI receiving tx for depression	N=100 (40 CBT-T; 18 CBT-IP; 42 UC)<br><br>CBT:<br>- Sex [Males]: 59%<br>- Age [Mean ± SD, Years]: 45.4 ± 14.1<br>- Race/ Ethnicity: Non-Hispanic White (90%)<br>- Marital Status: Single/Never Married (31%); Married/Partnered (19%); Divorced (41%); Separated (5%); Widowed (2%); Other (2%)<br>- Education: Some College (45%); Undergrad Degree (16%); Grad Degree (5%); Tech/Vocational (9%); High School Diploma (14%); ≤High School/GED (12%)<br><br>UC:<br>- Sex [Males]: 69%<br>- Age [Mean ± SD, Years]: 46.3 ± 12.4<br>- Race/ Ethnicity: Non-Hispanic White (90%)<br>- Marital Status: Single (29%); Married/Partnered (31%); Divorced (36%); Separated (5%)<br>- Education: Some College (45%); Undergrad Degree (17%); Grad Degree (17%); Tech/Vocational (5%); High School Diploma (5%); ≤High School/GED (12%)	CBT:<br>"complicated, mild/moderate" (69%); Severe (31%)/3.41 ± 2.84<br><br>UC: "complicated, mild/moderate" (69%); Severe (31%)/3.21 ± 2.58	Depression/NR<br><br>Other MHSU: Current Dysthymic (12%); Alcohol Abuse (10%); Substance Abuse (4%); hx of PTSD (18%)/NR	Treatment	CBT-T and CBT-IP<br>- adaptations to make protocol more accessible for people with cognitive impairment<br><br>UC:<br>- Notified by phone of depression status and encouraged to continue using rehabilitation and primary care services<br>- Free to self-refer to MHSU services outside of study<br>- Local MH resources provided to patient	Micro	Clinical	1.1	N/A	N/A	- Selection<br>- Nondifferential misclassification<br>- Ascertainment<br>- Recall

Boyd et al., 2016<br><br>United States	Case Study<br><br>Determine efficacy of CPT in tx of PTSD among individuals with hx of mTBI	N=1<br>- Sex: Male<br>- Age [Years]: "early 40s"<br>- Race/Ethnicity: Caucasian<br>- Marital Status: "Separated from wife"<br>- Education: High School + 1 year of College	mTBI/1 year prior to tx	PTSD/10+ years, sought care 5 mos post-TBI	Treatment	CPT-Cognitive Only	Micro	Clinical	1.1	N/A	N/A	- Selection<br>- Information<br>- Performance
Brenner et al., 2015<br><br>United States	Cohort<br><br>Screening for TBI among veterans seeking MH services and establish classification accuracy of TBI-4 using OSU-TBI as criterion standard	N= 316 (238 OSU TBI-ID pos; 78 OSU TBI-ID neg)<br><br>OSU TBI-ID pos:<br>- Sex [Males]: 92%<br>- Race/ Ethnicity: White (54%); African American (25%); Hispanic (13%); Other (7%)<br><br>OSU TBI-ID neg:<br>- Sex [Males]: 85%<br>- Race/ Ethnicity: White (62%); African American (22%); Hispanic (8%); Other (9%)	Severity of worst TBI:<br>Altered consciousness (22%); <5 min (35%); 5-30 min (16%); >30min (27%)<br><br>Time since first TBI: 33.2 ± 15.2	NR (all patients sought MH services)/NR	Screening	Screening for TBI using TBI-4 in MHSU setting	Meso	Clinical	4	N/A	N/A	N/A
Brown et al., 2019<br><br>United States	Cohort<br><br>Examine efficacy of neurofeedback therapy among IPV survivors w/TBI	N=32<br>- Sex [Males]: 3%<br>- Age [Mean, Years]: 46.9<br>- Ethnicity: White (63%); Hispanic (19%); Black/African American (13%); Asian (5%)	Moderate/NR	Depression; Anxiety; PTSD/NR	Treatment	Neurofeedback therapy	Micro	Clinical	1.1	N/A	N/A	- Selection<br>- Information<br>- Performance
Carrick et al., 2015<br><br>United States	Before-After, No Control<br><br>Evaluate effectiveness of vestibular rehabilitation to reduce PTSD sxs among individuals w/combat-related TBI	N=98<br>- Sex [Males]: 100%<br>- Age [Mean, Years]: 39	NR/NR	PTSD/NR	Treatment	Vestibular rehabilitation	Micro	Clinical	1.1	N/A	N/A	- Selection<br>- Performance
Carrick et al., 2015<br><br>United States	Before-After, No Control<br><br>Understand LT efficacy of vestibular rehabilitation in reducing PTSD sxs among individuals w/combat-related TBI	N=26<br>- Sex [Males]: 100%<br>- Age [Mean, Years]: 38.5	NR/NR	PTSD/NR	Treatment	Vestibular rehabilitation	Micro	Clinical	1.1	N/A	N/A	- Selection<br>- Performance

Cole et al., 2015<br><br>United States	Before-After, No Control<br><br>Explore safety, acceptability, feasibility, and efficacy of Mindfulness-Based Stress Reduction on neurocognitive and psychiatric measures in individuals w/PTSD and hx of mTBI	N=9<br>- Age [Mean ± SD, Years]: 45.6 ± 11.6<br>-Race/Ethnicity: White/ Non-Hispanic (33.3%); Hispanic/Latino (22.2%); African American (44.4%)	mTBI/ ≥12 months	PTSD/NR	Treatment	Mindfulness-Based Stress Reduction	Micro	Clinical	1.1	- Appointment reminder	N/A	- Information<br>- Selection<br>- Performance
D'Antonio et al., 2013<br><br>United States	RCT<br><br>Examine differential tx effects of CBT and SPT on self-reported sxs of depression in individuals w/TBI who sought tx for depression	N=44 (22 CBT; 22 SPT)<br>- Sex [Females]: 57.1%<br>- Age [Mean ± SD, Years]: 48.8 ± 10.2<br>- Race/Ethnicity: Caucasian (57%); Hispanic (20%); African American (15%)<br>- Education: ≤ High School (27%); Some College/College Degree (36%); Beyond College Degree (23%)	Mild (9%); Moderate (9.3%); Severe (15%)/7.69 (SD NR)	MDD/NR	Treatment	CBT:<br>- Modified to address cognitive deficits associated with TBI<br><br>SPT	Micro	Clinical	1.1	N/A	N/A	- Selection<br>- Information<br>- Ascertainment
Dindo et al., 2020<br><br>United States	Qualitative and RCT<br><br>Develop and refine ACT workshop and examine acceptability, feasibility and effect of ACT for veterans w/mTBI and MHSU	**Phase 1**<br>N= 11 Veterans (Workshop 1 = 5; Workshop 2 = 6)<br>- Gender [Men]: 100%<br>- Age [Mean ± SD, Years]: 37.5 ± 6.1<br>- Ethnicity: Hispanic (45%); African American (36%); White (18%)<br>- Marital Status: Currently Married (45%)<br>- Education [Mean ± SD, Years]: 14.3 ± 2.1<br>- Employment [Currently Employed]: (45%)<br><br>**Phase 2**<br>N= 32 (20 ACT; 12 UC)<br><br>ACT<br>- Age [Mean ± SD, Years]: 37.7 ± 6.3<br>- Ethnicity: White (42%); African American (21%); Hispanic/Latino (21%); Other (15%)<br>- Marital Status: 68%<br>- Education [Mean ± SD, Years]: 14.1 ± 1.8<br>- Employment: 47%<br><br>UC<br>- Age [Mean ± SD, Years]: 34.7 ± 5.8<br>- Ethnicity: White (42%); African American (17%); Hispanic/Latino (25%); Other (17%)<br>- Marital Status: 50%<br>- Education [Mean ± SD, Years]: 14.3 ± 1.6<br>- Employment: 58%	mTBI/NR (Phase I & II)	PTSD; Substance Dependence/NR	Treatment	Acceptance and Commitment Therapy<br>- Modified for veterans to include more veteran-centred language and experiences<br>- Clinical psychologist, neuropsychiatrist, cognitive psychologist, chaplain and anthropologist provided feedback on exercises and examples in development of protocol	Meso	Service	1.1<br>4.1	- Incorporating veteran into tx team<br>- Greater use of military terminology<br>- Increased time reviewing manuals and practicing learned skills during session<br>- Adjusting workshop timing	- Cognitive barriers to using patient manual and workshop skills (e.g., forgetting learned skills, misplacing manual)	- Selection<br>- Information<br>- Ascertainment
Eads et al., 2015<br><br>United States	Case Study<br><br>Alert hypnosis for PTSD sxs	N=1<br>- Sex: Male<br>- Marital Status: "Twice divorced"<br>- Education: "Minimal education"	NR/NR	PTSD/NR	Treatment	Alert Hypnotic Induction	Micro	Clinical	1.1	N/A	N/A	- Selection<br>- Information<br>- Performance<br>- Reporting

Elbogen et al., 2019<br><br>United States	RCT<br><br>Test effects of cognitive rehabilitation on executive function and emotion regulation in veterans w/TBI and PTSD	N=112 (55 Control; 57 CALM)<br><br>Control:<br>- Age [Mean ± SD, Years]: 36.3 ± 8.3<br>- Sex [Males]: 90%<br>- Race [Minority Status]: 50%<br><br>CALM:<br>- Age [Mean ± SD, Years]: 36.8 ± 8.6<br>- Sex [Males]: 90%<br>- Race [Minority Status]: 34%	Control: Moderate/Severe (53%)/NR<br><br>CALM: Moderate/Severe (61%)/NR	PTSD/NR	Treatment	Cognitive Rehabilitation (Cognitive Applications for Life Management)<br>- Included mobile app to break down goals into steps and to record steps and set reminders to complete action items<br><br>Control:<br>- Psychoeducation on TBI<br>- Mobile device to train visual memory	Micro	Clinical	1.1<br>3.5	N/A	N/A	- Selection<br>- Information
Elbogen et al., 2019<br><br>United States	Before-After, No Control<br><br>Examine feasibility of mobile neurofeedback therapy to manage pain in veterans w/TBI and PTSD	N= 36<br>- Age [Mean ± SD, Years]: 38.6 ± 10.0<br>- Sex [Males]: 85.4%<br>- Race: White (41.5%); Black (39.0%); American Indian (4.9%); Other (14.6%)<br>- Education [Mean ± SD, Years]: 13.6 ± 4.4	No LOC (30.8%); <1min LOC (20.5%); 1-15 min LOC (33.3%); 16-30 min LOC (7.7%); >30 min LOC (7.7%) / 13.1 ± 9.9	PTSD/NR	Treatment	Neurofeedback therapy<br>- Portable headset linked to an application on mobile device	Micro	Clinical	1.1	N/A	N/A	- Information<br>- Performance
Fann et al., 2015<br><br>United States	RCT<br><br>Test efficacy of CBT to tx depression w/in 10 years of complicated mild to severe TBI	N=100 (40 CBT-T; 18 CBT-IT; 42 UC)<br><br>CBT:<br>- Sex [Males]: 59%<br>- Age [Mean ± SD, Years]: 45.4 ± 14.1<br>- Race: Non-Hispanic White (90%)<br>- Education: Some College (53%); College Degree (21%); High School Diploma (14%); ≤GED (12%)<br>- Marital Status: Single (31%); Married/Partnered (19%); Divorced (41%); Separated (5%); Widowed (2%); Other (2%)<br><br>UC:<br>- Sex [Males]: 69%<br>- Age [Mean ± SD, Years]: 46.3 ± 12.4<br>- Race: Non-Hispanic White (90%)<br>- Education: Some College (50%); College Degree (33%); High School Diploma (5%); ≤GED (12%)<br>- Marital Status: Single (29%); Married/Partnered (31%); Divorced (36%); Separated (5%)	CBT: "Complicated mild/moderate" (69%); Severe (31%)/3.41 ± 2.84<br><br>UC: "Complicated mild/moderate" (69%); Severe (31%)/ 3.21 ± 2.58	Depression/NR	Treatment	CBT-T and CBT-IP:<br>- tailored to individuals with TBI<br><br>UC:<br>- Notified by phone of depression status and encouraged to continue using rehabilitation and primary care services available<br>- List of local MH and TBI resources provided to self-refer to MH services outside of study	Micro	Clinical	1.1	N/A	N/A	- Selection<br>- Information<br>- Performance

Fann et al., 2017<br><br>United States	RCT<br><br>Compare efficacy of pharmacotherapy for depression w/in 1 year of complicated mild to severe TBI	N=62 (31 Sertraline; 31 Placebo)<br><br>Sertraline:<br>- Age [Mean ± SD, Years]: 38.0 ± 12.3<br>- Sex [Males]: 74%<br>- Race: Non-Hispanic White (81%); Hispanic/Latino (6%); Non-Hispanic Black (6%); Asian/Pacific Islander (3%); Other (3%)<br>- Education [Completed High School]: 77%<br>- Marital Status: Never Married (35%); Married (35%); Divorced/Separated/Widowed (29%)<br><br>Placebo:<br>- Age [Mean ± SD, Years]: 36.9 ± 12.9<br>- Sex [Males]: 77%<br>- Race: Non-Hispanic White (81%); Hispanic/Latino (6%); Non-Hispanic Black (10%); Other (3%)<br>- Education [Completed High School]: 74%<br>- Marital Status: Never Married (45%); Married (19%); Divorced/Separated/Widowed (35%)	Sertraline: Complicated Mild (52%); Moderate (19%)/NR<br><br>Placebo: Complicated Mild (42%); Moderate (23%)/NR	Depression/NR	Treatment	Psychotherapy (Sertraline)	Micro	Clinical	1.1	N/A	N/A	- Selection<br>- Information<br>- Attrition
Gertler et al., 2019<br><br>Australia	Case Study<br><br>Investigate Behavioural activation therapy to improve activity participation and mood for individuals with depression after TBI	N=2<br>- Age [Years]: 26, 46<br>- Gender [Men]: 100%<br>- Marital Status: Separated<br>- Employment: Unemployed	Mild/2 years<br><br>Severe/1 year 3 mos	MDD (N=2); Alcohol Dependence (N=1)/NR	Treatment	Behavioural Activation Therapy	Micro	Clinical	1.1<br>3.5	N/A	N/A	- Selection<br>- Information<br>- Performance
Gros et al., 2017<br><br>United States	RCT<br><br>Examine influence of mTBI on Prolonged Exposure to treat PTSD and SUD	N=51 (30 TBI+LOC; 21 Controls)<br><br>TBI+LOC:<br>- Sex [Males]: 97%<br>- Age [Mean ± SD, Years]: 40.7 ± 11.1<br>- Race/Ethnicity: White (73%)<br>- Marital Status: Single (33%); Married (17%)<br>- Employment: Employed (36%)<br>- Education [Mean ± SD, Years]: 13.6 ± 2.1<br><br>Controls:<br>- Sex [Males]: 86%<br>- Age [Mean ± SD, Years]: 38.7 ± 10.4<br>- Race/Ethnicity: White (62%)<br>- Marital Status: Single (19%); Married (38%)<br>- Employment: Employed (55%)<br>- Education [Mean ± SD, Years]: 14.7 ± 2.1	mTBI/NR	SUD, PTSD/NR	Treatment	Prolonged Exposure (COPE - "Concurrent tx of PTSD and SUD using Prolonged Exposure")	Micro	Clinical	1.1	N/A	N/A	- Information<br>- Selection<br>- Performance<br>- Ascertainment

Haarbauer-Krupa et al., 2017<br><br>United States	Cohort<br><br>Examine incidence and factors associated w/PTSD 6 mos post-injury in civilian ED population	N=280 (75 w/PTSD; 205 w/out PTSD)<br><br>w/PTSD:<br>- Sex [Males]: 72%<br>- Age [Mean ± SD, Year]: 42.0 ± 14.9<br>- Race: Caucasian (73%)<br>- Marital Status: Single (59%); Married (20%); Separated/Divorced (16%)<br>- Education [Mean ± SD, Year]: 13.5 ± 2.9<br><br>w/out PTSD:<br>- Sex [Males]: 68%<br>- Age [Mean ± SD, Year]: 43.3 ± 18.8<br>- Race: Caucasian (85%)<br>- Marital Status: Single (51%); Married (35.1%); Separated/Divorced (6.3%)<br>- Education [Mean ± SD, Year]: 14.7 ± 2.8	w/PTSD: Minor/Moderate (76%); Moderate/Severe/Critical (24%)/6 months<br><br>w/out PTSD: Minor/Moderate (68%); Moderate/Severe/Critical (32%)/6 months	PTSD/NR	Screening	Screening for PTSD using PCL-C in ED	Meso	Clinical	4	N/A	N/A	N/A
Hart et al., 2020<br><br>United States	RCT<br><br>Assess Behavioural Activation Therapy and SMS motivational messages to promote MHSU in individuals w/TBI	N=49 (38 Intentional Condition [INT]; 21 Motivation Condition [MOT])<br><br>INT:<br>- Age [Mean ± SD, Years]: 40.4 ± 15.2<br>- Sex [Males]: 79%<br>- Education [Mean ± SD, Years]: 13.2 ± 2.2<br>- Race/Ethnicity [White]: 44.7%<br><br>MOT:<br>- Age [Mean ± SD, Years]: 38.5 ± 15.3<br>- Sex [Males]: 81%<br>- Education [Mean ± SD, Years]: 13.0 ± 1.8<br>- Race/Ethnicity [White]: 38%	INT: "At least complicated-mild injury severity"/ 7.4 ± 5.5<br><br>MOT: "At least complicated-mild injury severity"/ 4.4 ± 4.3	"At least mild depression and/or anxiety"/NR	Treatment	Behavioural Activation Therapy + SMS (text) messages:<br>- Implementation intentions supporting individualized goals for increased rewarding/meaningful activities (8 weeks)<br><br>Attention control session + SMS messages:<br>- Motivational SMS messages (8 weeks)	Micro	Clinical	1.1<br>3.5	N/A	N/A	- Information<br>- Selection<br>- Performance<br>- Ascertainment
Hofer et al., 2013<br><br>Switzerland	Case Study<br><br>Describe psychotherapy and pharmacotherapy for OCD following TBI	N=1<br>- Gender: Man<br>- Age: 27 years<br>- Education: 12 years<br>- Employment: Full-Time at time of injury	Severe/3 years	Daily marijuana use, daily cocaine use/Stopped consumption 1 year before TBI	Treatment	Psychotherapy (Prolonged Exposure, CBT)<br><br>Pharmacotherapy (Paroxetine)	Micro	Service	1.1<br>4.1	N/A	N/A	- Selection<br>- Information
Iliceto et al., 2018<br><br>United States	Case Study<br><br>Demonstrate safe and successful use of rTMS for depression in patient w/hx of severe TBI	N=1<br>- Age [Years]: 37	Severe/NR	Anxiety, Bipolar/NR	Treatment	rTMS	Micro	Clinical	1.1	N/A	N/A	- Selection<br>- Information<br>- Performance<br>- Reporting

Jak et al., 2019<br><br>United States	RCT<br><br>Compared efficacy of CPT with SMART-CPT for comorbid PTSD and TBI	N=100 (49 CPT; 51 SMART-CPT)<br><br>CPT:<br>- Age [Mean ± SD, Years]: 33.9 ± 7.3<br>- Sex [Males]: 87.8%<br>- Ethnicity: Caucasian (40.8%); African-American (16.3%); Asian (14.3%); Hispanic/Latino (20.4%); Native Hawaiian/Pacific Islander (4.1%); Native American/Native Alaskan (2.0%); Other (2.0%)<br><br>SMART-CPT:<br>- Age [Mean ± SD, Years]: 34.8 ± 8.5<br>- Sex [Males]: 90.2%<br>- Ethnicity: Caucasian (52.9%); African-American (9.8%); Asian (3.9%); Hispanic/Latino (25.5%); Native Hawaiian/Pacific Islander (3.9%); Native American/Native Alaskan (2.0%), Other (2.0%)	CPT:<br>Moderate (8.2%)/5.8 ± 4.0<br><br>SMART-CPT:<br>Moderate (3.9%)/ 4.9 ± 3.0	PTSD/NR	Treatment	SMART-CPT:<br>- All standard components and structures of CPT<br>- Adaptations made to mTBI and cognitive complaints	Micro	Clinical	1.1	N/A	N/A	- Information<br>- Performance<br>- Attrition<br>- Selection
Janak et al., 2017<br><br>United States	Before-After, No Control<br><br>Investigate pre- to post-tx changes in PTSD and persistent post-concussive sxs	N=257<br>- Sex [Males]: 89%<br>- Age: 18-24 years (29.6%); 25-29 years (22.2%); 30-37 years (26.0%); ≥38 years (22.2%)	Mild/Median = 5 months; IQR = 47-573 days	PTSD/NR	Program	Multidisciplinary Tx Program:<br>- Psychotherapy, pharmacotherapy, vestibular rehabilitation, musculoskeletal therapy<br>- OT, PT, medical personnel, psychologist, SLP	Micro	Service	1.1<br>4.1	N/A	N/A	- Selection<br>- Information<br>- Reporting<br>- Performance
Jones et al., 2019<br><br>United States	Case study<br><br>Describe military service personnel w/TBI and PTSD and describe participation in art therapy program	N=3<br>- Age [Years]: 23, 32, 31<br>- Gender [Men]: 100%	mTBI/NR	PTSD (N=3), Anxiety (N=2), Depression (N=1)/NR	Treatment	Stage-based art therapy	Micro	Clinical	1.1	- Empowerment (support from tx team, fellow group members, and community)	N/A	- Selection<br>- Information<br>- Performance<br>- Reporting
Jorge et al., 2016<br><br>United States	RCT<br><br>Assess efficacy of Sertraline in prevention depressive disorders following TBI	N=94 (48 Sertraline; 46 Placebo)<br><br>Sertraline:<br>- Sex [Males]: 54%<br>- Age [Mean ± SD, Years]: 50.0 ± 20.1<br>- Race: White (98%)<br>- Marital Status: Married (55%); Single (21%); Divorced (17%); Widowed (6%)<br>- Employment: Employed (72%); Retired (17%); FT student (4%); Disabled (2%); Unemployed (2%)<br>- Education [Mean ± SD, Years]: 13.5 ± 1.9<br><br>Placebo:<br>- Sex [Males]: 65%<br>- Age [Mean ± SD, Years]: 54.9 ± 18.2<br>- Race: White (98%)<br>- Marital Status: Married (62%); Single (16%); Divorced (11%); Widowed (11%)<br>- Employment: Employed (58%); Retired (33%); FT students (2%); Disabled (0%); Unemployed (2%)<br>- Education [Mean ± SD, Years]: 14.1 ± 2.6	Sertraline:<br>Mild (79%), Moderate (10%), Severe (10%)/NR<br><br>Placebo:<br>Mild (78%), Moderate (13%), Severe (9%)/NR	Mood Disorder	Treatment	Sertraline	Micro	Clinical	None	N/A	N/A	- Selection<br>- Attrition

Juengst et al., 2015<br><br>United States	Before-After, No Control<br><br>Describe development of smartphone application for assessing mood-related sxs after TBI and examine feasibility of using this in community-based sample of adults with TBI	N=20<br>- Sex [Males]: 60%<br>- Age [Mean ± SD, Years]: 36.7 ± 12.4<br>- Race: 95%<br>- Marital Status: Married (30%)<br>- Education [Mean ± SD, Years]: 15.0 ± 2.3	"Complicated mild to severe TBI"/ 5.2 ± 3.6	Depression; Anxiety/NR	Screening	iPerform (user-centred cross-platform smartphone application) for self-monitoring of depression and anxiety sxs	Meso	Clinical	3.5	- Simplicity w/application interface and question content and style<br>- Effective and efficient communication pathways b/t individuals and clinicians/researchers<br>- Proper training	- Redundant and complex language<br>- Alternating assessments (e.g., multiple scales for different questions, differing time frames of references for different questions) --> confusion	N/A
Jurick et al., 2020<br><br>United States	RCT<br><br>Determine whether individuals w/scores in the invalid vs. valid range of the performance validity test show similar benefits from psychotherapy and if psychotherapy improves performance validity test performance	N=100<br>- Age (Mean ± SD, Years): 34.4 ± 7.9<br>- Sex [Males]: 89%<br>- Race: Caucasian (70%)<br>- Education (Mean ± SD, Years): 13.7 ± 1.8	Mild, moderate/5.4 ± 3.5	PTSD/NR	Treatment	SMART-CPT:<br>- All standard components and structures of CPT<br>- Adaptations made to mTBI and cognitive complaints	Micro	Clinical	1.1	N/A	N/A	- Information<br>- Performance<br>- Attrition<br>- Selection
Kaimal et al., 2019<br><br>United States	Cross-sectional<br><br>Present findings on participant feedback on short- and long-term art therapy service for military service members w/PTSD and TBI	N=204<br>Sex: N=204 Males N=12 Females (as reported in study)	N=57 Unknown; N=47 Mild; N=20 Moderate; N=1 Severe (as reported in study)/NR	PTSD/NR	Treatment	Long-term art therapy program in outpatient integrative medical care facility<br>- Group and individual therapy	Meso	Clinical	1.1	N/A	N/A	- Information<br>- Reporting<br>- Selection<br>- Low survey completion rate
Kip et al., 2019<br><br>United States	Before-After, No Control<br><br>Present data for use of Accelerated Resolution Therapy among individuals with PTSD and TBI	N=202<br>- Age [Mean ± SD, Years]: 43.0 ± 12.8<br>- Sex [Males]: 90.1%<br>- Race: White (83.6%); Black (12.9%); Other (3.5%)<br>- Education [Mean ± SD, Years]: 14.5 ± 2.7	Mild (N=48); Moderate/Severe (N=49)/NR	PTSD/NR	Treatment	Accelerated Resolution Therapy:<br>- Relaxation and orientation<br>- Imaginal exposure<br>- Imagery rescripting<br>- Assessment and closeout	Micro	Clinical	1.1	N/A	N/A	- Selection<br>- Performance

Levy et al., 2019<br><br>United States	Qualitative<br><br>Examine the potential, usability, relevance, and acceptability of virtual reality grocery store as an assessment and intervention tool for veterans w/mTBI	N=6 focus groups<br>- Therapist: N=6 at initial, N=7 at follow-up<br>- Veterans w/mTBI and PTSD: N=3 at initial, N=4 at follow-up	mTBI/NR	PTSD/NR	Treatment	V-Mart<br>- Prototype of an interactive, navigable, fully stocked, 7-aisle virtual grocery store designed as a therapist guided tool for assessment and tx of cognitive and emotional impairments associated with mTBI and/or PTSD	Meso	Clinical	1.1<br>3.5	N/A	For therapist:<br>- Technical issues (difficult to use if not skilled w/game playing)	- Small sample
Luo et al., 2015<br><br>China	Cohort<br><br>Investigate relationship between degree of depression in patients w/TBI and severity and progression of TBI; therapeutic effects of Prednisone w/psycho-interventions and anti-depression drugs	N=68<br>- Sex [Males]: 66%<br>- Age [Years]: 18 to 70	All/NR	Depression/"observed from 3 mos - 3 years after TBI"	Treatment	Pharmacotherapy (Citalopram, Prednisone)<br><br>Psychotherapy<br>- Interpersonal therapy, behavioural activation, CBT, problem solving therapy, social skills training, psychodynamic therapy, supportive counseling	Micro	Service	1.1<br>4.1	N/A	N/A	- Selection<br>- Performance<br>- Information
Moss, 2018<br><br>United States	Case Study<br><br>Describe integration of hypnosis and variety of palliative care strategies into multi-modal tx program	N=1<br>- Gender: Woman<br>- Age [Years]: 38<br>- Marital Status: Married<br>- Employment: Formerly employed	NR/2 years	Depression; PTSD/NR	Treatment	Hypnosis, energy therapy, psychotherapy, acupuncture, medication management	Micro	Service	1.1<br>4.1	N/A	N/A	- Selection<br>- Information<br>- Reporting<br>- Performance
O'Connor et al., 2016<br><br>United States	RCT<br><br>Evaluate 12 week cognitive rehabilitation intervention embedded w/vocational rehabilitation services for veterans w/hx of mTBI and MHSU	N=18 (10 cog rehab; 8 control)<br>- Sex [Males]: 100%<br>- Age [Mean ± SD, Years]: 51.0 ± 8.6<br>- Ethnicity: White (61.1%); Hispanic/Latino (22.2%); African-American/Black (16.7%)<br>- Education [Mean ± SD, Year]: 13.0 ± 1.7; High School Diploma (83.3%); Associate's Degree (11.1%); Bachelor's Degree (5.6%)	mTBI/NR	PTSD (N=8); MDD (N=5); Bipolar (N=3); OCD (N=1); Schizoaffective Disorder (N=1); Generalized anxiety disorder; (N=1); Other anxiety and mood disorder; (N=7); Alcohol Abuse/Dependence (N=9); Opiate Abuse/Dependence (N=2); Cocaine Abuse (N=2) /NR	Treatment	Cognitive rehabilitation<br>- Compensatory strategies to manage cognitive deficits<br>- Laptop w/software to address training and rehabilitation needs<br>- veteran + cognitive rehabilitation specialist met with vocational rehabilitation specialist	Meso	Service	1.1<br>3.5<br>4.1	- Lack of uniformity in module across exercises<br>- Expanded word count (for computer response)	- Computer software (navigation that requiring restructuring of elements on screen, limited word count)<br>- Remembering password	- Selection<br>- Information<br>- Performance<br>- Ascertainment<br>- Reporting

Olson-Madden et al., 2014<br><br>United States	Cohort<br><br>Determine whether a positive screen on TBI-4 can be used to identify veterans who use more inpatient and outpatient MH services	AnyQ+/-: Any question positive/negative; Q2+/-: Question 2 positive/negative<br>N=1,493 (AnyQ+: 942; AnyQ-: 551; Q2+: 657; Q2-: 836)<br><br>AnyQ+: - Sex [Males]: 91.9%<br>- Age [Mean ± SD, Years]: 47.6 ± 13.0<br>- Race/Ethnicity: White (74.5%); Black (11.7%); Hispanic (0.9%); Other (2.8%); Missing (10.2%)<br>- Marital Status: Married (28.1%); Divorced/Separated/Widowed (46.8%); Single (24.2%); Missing/unknown (0.9%)<br><br>AnyQ-: - Sex [Males]: 88.0%<br>- Age [Mean ± SD, Years]: 47.8 ± 13.7<br>- Race/Ethnicity: White (70.4%); Black (17.2%); Hispanic (0.5%); Other (2.2%); Missing (9.6%)<br>- Marital Status: Married (36.7%); Divorced/Separated/Widowed (39.9%); Single (23.2%); Missing/Unknown (0.2%)<br><br>Q2+: - Sex [Males]: 94.7%<br>- Age [Mean ± SD, Years]: 47.6 ± 13.1<br>- Race/Ethnicity: White (78.1%); Black (9.6%); Hispanic (0.6%); Other (2.0%); Missing (9.7%)<br>- Marital Status: Married (28.9%); Divorced/Separated/Widowed (46.3%); Single (24.2%); Missing/Unknown (0.6%)<br><br>Q2-: - Sex [Males]: 87.2%<br>- Age [Mean ± SD, Years]: 47.8 ± 13.4<br>- Race/Ethnicity: White (69.0%); Black (17.0%); Hispanic (0.8%); Other (3.0%); Missing (10.2%)<br>- Marital Status: Married (33.1%); Divorced/Separated/Widowed (42.7%); Single (23.6%); Missing/Unknown (0.6%)	NR/NR	NR (all patients sought MH services)/NR	Screening	Screening of TBI using TBI-4	Meso	Clinical	4	N/A	N/A	N/A
Ponsford et al., 2019<br><br>Australia	RCT<br><br>Investigate factors associated w/positive response to adapted CBT intervention for individuals w/anxiety and depression following TBI	Motivational Interviewing (MI); Non-directive counseling (NDC)<br>N=45 (22 MI + CBT; 23 NDC + CBT)<br><br>MI+CBT:<br>- Age [Mean ± SD, Years]: 47.7 ± 16.1<br>- Sex [Males]: 92.7%<br>- Education [Mean ± SD, Years]: 13.7 ± 3.5<br><br>NDC+CBT:<br>- Age [Mean ± SD, Years]: 40.4 ± 13.4<br>- Sex [Males]: 73.9%<br>- Education [Mean ± SD, Years]: 12.9 ± 3.1	MI+CBT: GCS [Mean ± SD]; 10.7 ± 3.7/ 5.6 ± 12.3<br><br>NDC+CBT: GCS [Mean ± SD]; 10.2 ± 4.3/ 3.8 ± 6.2	Anxiety; Depression/NR	Treatment	CBT + Motivational interviewing (to enhance motivation and tx self-efficacy)<br><br>CBT + Nondirective counseling<br><br>CBT adapted for cognitive impairments	Micro	Clinical	1.1	N/A	N/A	- Selection<br>- Information<br>- Ascertainment<br>- Performance
Ragsdale et al., 2016<br><br>United States	Cohort Study<br><br>Examine effectiveness of completing prolonged exposure or CPT among veterans w/dx of PTSD with and w/out hx of TBI	N=41 (21 Prolonged Exposure; 20 CPT)<br>- Sex [Males]: 87.8%<br>- Age [Mean ± SD, Years]: 33.1 ± 8.2<br>- Race: Caucasian (85.4%); African American (7.3%); Hispanic/Latino (7.3%)<br>- Marital Status: Never married (32.5%); Married (26.8%); Divorced (17.1%); Remarried (9.8%); Separated (9.8%); Living w/partner (2.4%); Unknown (2.4%)	NR/NR	PTSD/NR	Treatment	CPT<br><br>Prolonged exposure therapy	Micro	Clinical	1.1	N/A	N/A	- Selection<br>- Information<br>- Performance<br>- Reporting

Ragsdale et al., 2018<br><br>United States	Cross-sectional<br><br>Compared the exposure therapy process in veterans w/PTSD alone to those w/PTSD and TBI hx	N=88 (45 TBI + PTSD; 43 PTSD only)<br><br>TBI+PTSD:<br>- Sex [Males]: 95.6%<br>- Age [Mean ± SD, Years]: 36.3 ± 8.7<br>- Race: Caucasian (68.9%); African-American (15.6%); Hispanic (13.3%); Other (2.2%)<br>- Education: High School (42.2%); Some College (31.1%); Bachelors (15.6%); Masters/Doctoral (11.1%)<br>- Marital Status: Married (57.8%); Single (13.3%); Separated (17.8%); Divorced (11.1%)<br><br>PTSD Only:<br>- Sex [Males]: 93.0%<br>- Age [Mean ± SD, Years]: 39.4 ± 9.7<br>- Race: Caucasian (67.4%); African-American (7.0%); Hispanic (14.0%); Asian (2.3%); Indian Subcontinent (2.3%); Other (7.0%)<br>- Education: High School (23.3%); Some College (53.5%); Bachelors (20.9%); Masters/Doctoral (2.3%)<br>- Marital Status: Married (46.5%); Single (20.9%); Separated (20.9%); Divorced (11.6%)	NR/NR	PTSD/NR	Treatment	Virtual reality assisted exposure therapy + group therapy	Micro	Clinical	1.1<br>3.5	N/A	N/A	- Information<br>- Nondifferential misclassification<br>- Selection<br>- Performance
Rao et al., 2019<br><br>United States	RCT<br><br>Acquire data to inform safety and tolerability of low-frequency right-sided rTMS in patients w/TBI and effective size over the dorsolateral prefrontal cortex on TBI depression and common comorbid psychiatric sxs	N=30 (17 Sham, 13 rTMS)<br><br>rTMS:<br>- Age [Mean ± SD, Years]: 39.8 ± 14.2<br>- Sex [Males]: 38%<br>- Race [Caucasian]: 69%<br>- Education [Mean ± SD, Years]: 14.6 ± 3.0<br>- Marital Status [Married/Partner]: 38%<br><br>Sham:<br>- Age [Mean ± SD, Years]: 40.2 ± 14.5<br>- Sex [Males]: 65%<br>- Race [Caucasian]: 59%<br>- Education [Mean ± SD, Years]: 14.4 ± 2.6<br>- Marital Status [Married/Partner]: 35%	rTMS:<br>Mild (100%)/3 mos- 1 year (23%); 1-5years (38%); 5-10 years (23%); >10 years (15%)<br><br>Sham: Mild (88%); Severe (12%)/ 3 mos-1 year (53%); 1-5 years (12%); 5-10 years (29%); >10 years (6%)	Shame: Depression/39.4 ± 43<br><br>rTMS: Depression/39.6 ± 36.1	Treatment	rTMS	Micro	Clinical	1.1	N/A	N/A	- Selection
Ravid et al., 2019<br><br>Israel	Case Study<br><br>Present case study in which integrative approach combined psychiatric medicine and traditional Chinese medicine	N=1<br>- Gender: Woman<br>- Age [Years]: 55<br>- Marital Status: Married<br>- Employment: Previously employed	NR/1 year	Anxiety, patterns of avoidance, introversion, difficulty expressing herself, anhedonia (inability to feel pleasure) and difficulty interacting w/people/NR	Treatment	Pharmacotherapy, acupuncture, herbal formula	Micro	Service	1.1<br>4.1<br>4.3	N/A	- Financial limitations	- Selection<br>- Information<br>- Reporting<br>- Performance

Roche et al., 2020<br><br>United Kingdom	Case Study<br><br>Illustrate management of PTSD in context of TBI using Acceptance and Commitment Therapy-based approach	N=1<br>- Gender: Woman<br>- Age [Years]: 48	Moderate/NR	PTSD/NR	Treatment	Acceptance and Commitment Therapy	Micro	Clinical	1.1	N/A	N/A	- Selection<br>- Information
Schneider et al., 2016<br><br>United States	Cohort<br><br>Determine whether a positive screen on TBI-4 is associated w/increased risk for suicide attempt w/in 1 year post-screening	N=1,097 (489 Positive; 629 Negative)<br><br>Positive:<br>- Sex [Males]: 94.4%<br>- Age [Mean ± SD, Years]: 47.8 ± 12.9<br>- Race/ Ethnicity: Caucasian (76.9%); African American (10%); Hispanic (0.6%); Other (1.9%); Missing (10.5%)<br>- Marital Status: Married (30.8%); Divorced/Separated/Widowed (45.5%); Single (22.9%); Missing/Unknown (0.9%)<br><br>Negative:<br>- Sex [Males]: 87.6<br>- Age [Mean ± SD, Years]: 48.2 ± 13.6<br>- Race/ Ethnicity: Caucasian (68%); African-American (17.2%); Hispanic (0.5%); Other (3.0%); Missing (11.3%)<br>- Marital Status: Married (35.5%); Divorced/Separated/Widowed (40.7%); Single (23.1%); Missing/Unknown (0.8%)	NR/NR	NR (all patients sought MH services)/NR	Screening	Screening for TBI using TBI-4 by MH clinicians during MH intake	Meso	Clinical	4	N/A	N/A	N/A
Siddiqi et al., 2019<br><br>United States	Case Study<br><br>Describe case in which resting state network mapping was successfully used to target rTMS to tx neuropsychiatric disturbances in retired NFL defensive linesman	N=1<br>- Gender: Man<br>- Age: "Fourth decade of life"	NR/NR	Depression; Anxiety; Impulsivity; Anger/"2-3 year hx"	Treatment	rTMS	Micro	Clinical	1.1	N/A	N/A	- Selection<br>- Information
Sripada et al., 2013<br><br>United States	Cohort (Study 1); RCT (Study 2)<br><br>Assess impact of prolonged exposure for individuals with and w/out hx of TBI	Study 1:<br>N=51<br>- Sex: NR<br>- Age [Mean ± SD, Years]: 49.3<br>- Race: White (96%); Black (4%)<br><br>Study 2:<br>N=22<br>- Sex [Females]: 9%<br>- Age [Mean ± SD, Years]: 32.7 ± 6.9<br>- Race: White (73%); African American (23%); Asian (5%)	mTBI/NR	Study 1:<br>PTSD; SUD (17%); Comorbid Psychiatric (64%)/NR<br><br>Study 2: PTSD; Depression (57%)/NR	Treatment	Prolonged exposure	Micro	Clinical	1.1	N/A	N/A	- Information<br>- Nondifferential misclassification<br>- Reporting<br>- Ascertainment<br>- Performance

Strom et al., 2016<br><br>United States	Case Study<br><br>Demonstrate successful implementation of prolonged exposure in instances where the presence of TBI-related cognitive and behavioural deficits interfered w/standard tx implementation	N=2<br>- Gender: Man (N=1); Woman (N=1)<br>- Age [Years]: 29 and 55<br>- Race/Ethnicity: Caucasian<br>- Marital Status [Married]: 100%	Severe (N=1), moderate (N=1)/NR	PTSD/NR	Treatment	Prolonged exposure therapy<br>- Compensatory strategies to account for cognitive deficits	Micro	Clinical	1.1	- Psychoeducation re: overlap of PTSD and TBI sxs<br>- Protocol-consistent modifications to address cognitive and behavioural deficits associated with TBI	N/A	- Selection<br>- Information<br>- Performance
Walker et al., 2016<br><br>United States	Case Study<br><br>Present therapeutic process for senior military service member who struggled w/untreated sxs of PTSD and moderate TBI	N=1<br>- Gender: Man<br>- Age [Years]: "50s"<br>- Marital Status: Married	Moderate/ "almost 7 years"	PTSD/NR	Treatment	Guided art therapy and acupuncture	Micro	Service	1.1	N/A	N/A	- Selection<br>- Information<br>- Reporting<br>- Performance
Whiting et al., 2020<br><br>Australia	RCT<br><br>Investigate if Acceptance and Commitment Therapy can facilitate psychological adjustment and reduce psychological stress following severe TBI	N=19 (10 ACT; 9 Control)<br><br>ACT:<br>- Age [Mean ± SD, Years]: 36.4 ± 13.5<br>- Sex [Males]: 80%<br>- Education [Mean ± SD, Years]: 11.2 ± 2.0<br><br>Control:<br>- Age [Mean ± SD, Years]: 37.2 ± 12.5<br>- Sex [Males]: 77.8%<br>- Education [Mean ± SD, Years]: 11.4 ± 1.0	ACT: Severe/20.7 ± 17.5<br><br>Control: Severe/33.3 ± 21.5	Depression; Anxiety; Stress/NR	Treatment	Acceptance and Commitment Therapy<br>- Strategies implemented to accommodate for cognitive impairment<br><br>Befriending therapy (Control):<br>- Therapy focuses on neutral topics that are unlikely to elicit a negative emotional response<br>- Relationship with participant is friendly an engaging rather than empathic<br>- Therapist provides positive statements rather than problem solving	Micro	Clinical	1.1	N/A	N/A	- Selection<br>- Information
Winter et al., 2016<br><br>United States	RCT<br><br>Test Veteran's In-Home Program to promote community re-integration, mitigate TBI sxs, and facilitate daily functioning	N=81<br>- Age [Mean ± SD, Year]: 40.1 ± 13.0<br>- Sex [Males]: Intervention (87.8%); Control (95%)<br>- Race: White (58.0%); Black (35.8%); Native American (1.2%); Asian (1.2%); No Primary (1.2%); Other (2.5%)<br>- Marital Status: Married (71.6%)<br>- Employment: Employed (34.6%)<br>- Education: <High School Degree (4.9%); 45.7% Some College Education (45.7%); 24.7% High School Degree/GED (24.7%); College Degree (16%); Post-Doc Degree (7.4%)	Intervention: Mild (70%)/ 9.98 ± 11.1<br><br>Control: Mild (58.3%)/ 9.48 ± 11.2	PTSD; Depression/NR	Program	Veteran's In-Home Program:<br>- OT meets with veteran and family to identify concerns, review intervention goals, introduce strategies for goals, home modification	Meso	Clinical	1.1<br>4.1	- Family involvement --> supports tx adherence and maintenance and provides education and support to family	N/A	- Selection<br>- Performance<br>- Information

Wolf et al., 2018<br><br>United States	Before-After, No Control<br><br>Examine generalization of the effectiveness of prolonged exposure therapy for PTSD in improving post-concussive sxs and other outcomes in military service members and veterans w/hx of mild to severe TBI	N=44<br>- Sex [Males]: 93.2%<br>- Age [Mean ± SD, Years]: 33.2 ± 7.34<br>- Ethnicity: Caucasian (70.5%); Hispanic (18.2%); African American (9.1%); Other (2.3%)<br>- Marital Status: Married (50%); Single (38.6%); Separated/Divorced (11.4%)	Mild (65.9%); Moderate/Severe (34.1%)/ 4.42 ± 2.99	PTSD; Depression (88.6%); Other Anxiety (27.3%); Substance Abuse (29.5%); hx of Suicide (29.5%)/ NR	Treatment	Prolonged exposure<br>- Modifications to accommodate TBI-related deficits	Micro	Clinical	1.1	- Modifications to accommodate TBI-related deficits<br>- Compensatory devices to provide support and corrective instruction<br>- "teach-back" method to ensure understanding of content and instructions<br>- recorded sessions and patient handouts	N/A	- Selection<br>- Performance<br>- Reporting
Yount et al., 2013<br><br>United States	Case Study<br><br>Demonstrate effects of service dog training as purpose-driven intervention on PTSD sxs and mTBI	N=2<br>- Gender [Men]: 100%	NR/NR	PTSD/NR	Treatment	Training of service dogs for fellow veterans	Micro	Clinical	1.1	N/A	N/A	- Selection<br>- Information<br>- Reporting<br>- Performance
Zhang et al., 2017<br><br>China	RCT<br><br>Compare effect of methylphenidate w/placebo in patients with TBI in China	N=36 (18 Intervention; 18 Control)<br><br>Intervention:<br>- Age [Mean ± SD, Years]: 36.3 ± 10.9<br>- Sex [Males]: 72.2%<br>- Ethnicity: Han (66.7%); Hui (33.3%)<br>- Education: Elementary School (22.2%); High School (44.4%); College/University (33.3%)<br><br>Control:<br>- Age [Mean ± SD, Years]: 34.9 ± 12.1<br>- Sex [Males]: 77.8%<br>- Ethnicity: Han (55.6%); Hui (44.4%)<br>- Education: Elementary School (33.3%); High School (33.3%); College/University (33.3%)	Mild-moderate/NR	Major depressive episode/NR	Treatment	Methylphenidate	Micro	Clinical	1.1	N/A	N/A	- Selection<br>- Information

ACT: Acceptance and Commitment Therapy; **ADHD:** Attention deficit hyperactivity disorder; **B/t:** between; **CBT:** Cognitive behavioural therapy; **CBT-T:** Cognitive behavioural therapy - telephone; **CBT-IP:** Cognitive behavioural therapy - in person; **CPT:** Cognitive processing therapy; **Dx:** Diagnosis; **ED:** Emergency department; **GCS:** Glasgow Coma Scale; **Gp:** Group; **HCP:** Healthcare professional; **Hx:** History; **IC:** Integrated care; **LOC:** Loss of consciousness; **LT:** Long-term; **MCBT:** Mindfulness based cognitive behavioural therapy; **MDD:** Major depressive disorder; **MH:** Mental health; **MHSU:** Mental health and/or substance use; **Mos:** Months; **mTBI:** Mild traumatic brain injury; **N/A:** Not applicable; **NR:** Not reported; **OCD:** Obsessive compulsive disorder; **OSU-OT:** Occupational therapy; **TBI-ID:** Ohio State University Traumatic Brain Injury Identification Method; **PT:** Physiotherapy; **PTSD:** Post-traumatic stress disorder; **QoL:** Quality of life; **rTMS:** Repetitive transcranial magnetic stimulation; **RCT:** Randomized controlled trials; **SD:** Standard deviation; **SLP:** Speech language pathology; **SPT:** Supportive psychotherapy; **SUD:** Substance use disorder; **Sxs:** Symptoms; **TBI:** Traumatic brain injury; **UC:** Usual care; **Tx:** Treatment**; W/:** With
